# Full-length RNA sequencing and single-nucleus sequencing deciphers programmed cell death and developmental trajectories in laticiferous canals of *Decaisnea insignis* fruits

**DOI:** 10.3389/fpls.2024.1446561

**Published:** 2024-08-13

**Authors:** Gen Li, Qian Zhao, Xinwei Shi, Bin Li, Luyao Yang, Yanwen Wang, Yafu Zhou

**Affiliations:** ^1^ Xi’an Botanical Garden of Shaanxi Province, Institute of Botany of Shaanxi, Xi’an, China; ^2^ Shaanxi Engineering Research Centre for Conservation and Utilization of Botanical Resources, Xi’an Botanical Garden of Shaanxi Province (Institute of Botany of Shaanxi), Xi’an, China; ^3^ Shaanxi Key Laboratory of Qinling Ecological Security, Xi’an Botanical Garden of Shaanxi Province (Institute of Botany of Shaanxi), Xi’an, China; ^4^ College of Biology Pharmacy and Food Engineering, Shangluo University, Shangluo, China

**Keywords:** snRNA-seq, full-length RNA seq, *Decaisnea insignis*, fruit development, programmed cell death (PCD)

## Abstract

**Introduction:**

Programmed cell death (PCD) is a fundamental biological process crucial for plant development. Despite recent advancements in our understanding of PCD’s molecular mechanisms, the intricate orchestration of this process within plant cells remains enigmatic. To address this knowledge gap, the present study focuses on *Decaisnea insignis*, a plant species renowned for its unique fruit anatomy, including laticiferous canals that secrete latex. While extensive anatomical studies have elucidated the structural features of these canals,molecular insights into their developmental regulation, particularly the involvement of PCD, are lacking.

**Methods:**

In this study, we sequenced the single-cell transcriptomes at two developmental stage of *Decaisnea insignis* fruit using the technology known as 10x Genomics (S1, S2). Using sequencing technology combining full- length RNA sequencing and single-nucleus RNA sequencing (snRNA-seq) in combination with ultrastructural analyses, our study revealed a cellular map of *Decaisnea insignis* fruit at the single-cell level and identified different cell types.

**Results:**

In particular, we identified a possible PCD-mediated cluster of *Decaisnea insignis* fruit lactiferous canals in epidermal cells and clarified the expression patterns of *DiRD21A* (a hydrolase) and *DiLSD1* (a transcription factor), which may be closely related to the development of laticiferous canals in *Decaisnea insignis* fruits.

**Discussion:**

By integrating high-resolution gene expression profiling with visual insights into cellular transformations, we sought to more precisely characterize the regulatory role of PCD during the developmental formation of lactiferous canals in *Decaisnea insignis* fruit.

## 1 Introduction

Programmed cell death (PCD) is a fundamental biological phenomenon integral to the lifecycles and adaptations of plants (Xie et al., 2022). Thus, understanding PCD is pivotal to understanding plant development, in which this process orchestrates the selective elimination of cells, thereby sculpting plant morphology and facilitating tissue differentiation. PCD in plants is a meticulously controlled process. This form of cellular self-destruction is activated during developmental processes such as cellular differentiation (Kwon et al., 2010), tissue senescence (Lee and Chen, 2002), and organ development (Wang et al., 2021). In particular, PCD plays a crucial role in the development of secretory structures in plants, including the mucilage cells of *Araucaria angustifolia* (Mastroberti and Mariath, 2008), floral nectary *of Digitalis purpurea* (Gaffal et al., 2007), *Gossypium hirsutum* pigment glands (Liu et al., 2010), secretory cavities of *Citrus sinensis* (Chen and Wu, 2010), secretory cavities and capitate glandular hairs in *Dictamnus dasycarpus* (Zhou et al., 2012; Zhou et al., 2023a), and laticiferous canal of *Decaisnea insignis* (Zhou and Liu, 2011; Zhou et al., 2023b). Advances in plant science research have elucidated components of the elaborate molecular machinery that drives PCD in organ development, including a cadre of proteases, nucleases, and signaling molecules (Zhang et al., 2014). These elements are regulated via an intricate network of genetic controls involving gene clusters implicated in life-and-death decisions and a multitude of signaling molecules (Zhang et al., 2023). As we further explore the regulatory networks of plants, it becomes increasingly clear that PCD is less of an isolated event and more an expression of the complex biological activity of cellular activities during the development of secretory structures. Therefore, new techniques are needed to more precisely elucidate the changes that occur in this biological activity.

The landscape of plant cell research has been dramatically altered by the advent of single-nucleus RNA sequencing (snRNA-seq) technology, a method that has empowered scientists to unravel the complexities of gene expression with single-cell precision (Denyer et al., 2019). This technological leap has facilitated an unprecedented refinement in our understanding of the spatiotemporal dynamics within individual cells (Li et al., 2023; Sun et al., 2023). Notably, snRNA-seq provides a high-resolution window into the transcriptional landscape, capturing the nuances and variations in gene expression that were previously obscure in bulk RNA sequencing approaches (Linnarsson and Teichmann, 2016). A recent study used snRNA-seq technology to determine the developmental trajectory of the salt gland in the salt-secreting saline plant *Limonium bicolor* and the gene expression signatures associated with decisions governing the developmental fate of this gland (Zhao et al., 2024). The application of snRNA-seq technology could be particularly transformative for studying the development of plant secretory structures. By enabling a granular examination of cell populations, snRNA-seq may be able to illuminate the transcriptional shifts that underpin the induction and execution of PCD, thus delivering insights into the molecular signposts that drive this programmed cell death and the relationship between signal-driven PCD and the development of secretory structures.

*Decaisnea insignis*, a member of the Lardizabalaceae family, presents an intriguing subject of analysis due to its unusual fruit anatomy, most notably the laticiferous canals widely distributed on the surface of the fruit. These canals are integral to the fruit’s structure and specialized in secreting latex—a feature that not only affords fascinating properties to its secondary metabolites but has also yielded significant interest in the corresponding developmental processes (Zhou and Liu, 2011; Zhou et al., 2023b). The literature to date has explored various aspects of *Decaisnea insignis.* For example, anatomical studies have mapped the plant’s laticiferous canal structures, finding that PCD cytologically mediates the development of laticiferous canals (Zhou and Liu, 2011). Additionally, there has been interest in exploring the relationship between the plant’s secondary metabolites and the phenomenon of PCD during development (Zhou et al., 2023b). However, while these studies have laid foundational knowledge, they have not closed the gap in our comprehension of the molecular level in the programmatic orchestration of laticiferous canal development, particularly the role of programmed cell death.

This analysis is grounded in the hypothesis that PCD orchestrates the configuration of secretory structures within the laticiferous canals of *Decaisnea insignis* during fruit development. By employing full-length RNA Seq and snRNA-seq technology, the present study aims to delineate the transcriptomic architecture underlying the occurrence of the PCD phenomenon corresponding to different stages of fruit development and associate these stages with specific alterations in the cellular ultrastructure. The analysis of snRNA-seq data within an ultrastructural context aims not only to anchor transcriptomic insights in visible cellular transformations but also to pioneer an analytical paradigm capable of demystifying the complexities of organ development at the single-cell level.

## 2 Materials and methods

### 2.1 Plant materials

*Decaisnea insignis* (Griffith) J. D. Hooker et Thomson is a perennial shrub. The samples from two stages (S1 stage: diameter 1 mm; S2 stage: diameter 2.5 mm) of *Decaisnea insignis* ovary and fruit were collected from the Qinling Mountains, Ningshan, Shannxi, China (33.58656356N, 108.43033283E).

### 2.2 Light microscopy

The samples from two stages were first divided into small pieces of approximately 1 mm^3^ and then pre-fixed in 2.5% glutaraldehyde in a 0.1 M phosphate buffer (pH 7.0) for 48 hours at 4°C. Subsequently, the samples were fixed with 1% osmic acid in the same phosphate buffer overnight. After three rinses with phosphate buffer (each lasting 30 minutes), the samples were dehydrated using a gradient series of alcohol, as follows: 30%, 40%, 50%, 70%, 85%, and 95% each time, followed by 100% twice, with each step lasting 30 minutes. Next, the samples were treated with propylene oxide and embedded in Epon 812 resin (Zhou et al., 2023b). Semi-thin sections (2 μm) were obtained using a Reichert-Jung ultramicrotome and then stained with toluidine blue O. Examination and documentation were performed using a Leica microscope (DMLB) equipped with a video camera (DFC 7000T; Wetzlar, Germany).

### 2.3 Scanning electron microscopy

Samples of *Decaisnea insignis* fruit at two developmental stages were pre-fixed with 2.5% glutaraldehyde in a 0.1 M phosphate buffer (pH 7.0) for 48 hours, followed by post-fixing with 1% osmium tetroxide overnight at 4°C. The samples were then dehydrated using a graded series of ethanol, treated with tertiary-butyl alcohol twice, and subsequently stored at -20°C overnight before being subjected to 24 hours of vacuum drying. The dried samples were mounted on slides, coated with gold for 1 minute using a Hitachi E-102 Ion sputter (Hitachi High-Technologies Corporation, Tokyo, Japan) and observed using a Hitachi TM-1000 tabletop scanning electron microscope (Hitachi, Tokyo, Japan).

### 2.4 Full-length RNA-seq

The full-length RNA-seq was performed using Gene Denovo (Guangzhou, China). In accordance with the manufacturer’s instructions, 5 mg of RNA was combined for each library after separately extracting RNA from each tissue using an RNAprep Pure plant kit (DP441, TIANGEN, Beijing, China). Following the instructions provided by the manufacturer (Clontech SMARTER cDNA synthesis kit), first- and second-strand cDNA were synthesized from polyA mRNA using Oligo-dT primers. We used the Pacific Biosciences DNA Template Prep Kit 2.0 for SMRT bell library preparation, and for SMRT sequencing, a Pacific Bioscience Sequel System was employed. The SMRTlink program (version 5.1) was utilized to generate the circular consensus sequence (CCS) from the original sequence data (Gordon et al., 2015). Next, based on poly (A) tails and 3’ and 5’ adapters, CCS was distinguished as both full-length and non-full-length reads. The pairwise alignments of full-length reads and similar FLNC (full-length non-chimeric) reads were hierarchically clustered using Minimap2 to obtain Unpolished Consensus Isoforms (UCIs). Then, consistent sequences were further corrected using the Quiver algorithm, and high-quality isoforms (HQ isoforms, prediction accuracy ≥ 0.99) were obtained based on the accuracy of the output sequences. Complete transcripts were annotated using BLASTx (Scott and Madden, 2024) searches against the National Center for Biotechnology Information non-redundant (Nr), Swissprot, and EuKaryotic Orthologous Groups (KOG) databases. Gene Ontology (GO) annotations, Kyoto Encyclopedia of Genes and Genomes (KEGG) orthology, and pathway annotations were used to categorize the functions.

### 2.5 Single-nucleus RNA sequencing

Sample preparation, nucleus isolation, library preparation, and sequencing were performed using a device from Gene Denovo Biotechnology Co., Ltd. (Guangzhou, China) following the guidelines of 10×Genomics (10×Genomics, Pleasanton, CA, USA). Briefly, we collected fruits from wild *Decaisnea insignis* at two different developmental stages, rinsed the fruits with saline, and snap-froze them with liquid nitrogen to minimize changes in gene expression. This process was immediately followed by the isolation of crude nuclei. A Countess^®^ II Automated Cell Counter was used to count cell nuclei and perform nucleus quality control with 0.4% Trypan blue. Then, the collected nuclei were diluted to 1000 nucleus/μL. Next, gel beads containing the barcode information were conjugated to the nucleus and enzyme mixture to form GEMs (Gel Beads-In-Emulsions). The gel beads were then lysed to release a capture sequence containing the barcode sequence. Next, we reverse-transcribed the cDNA fragments and added 10X barcode for the samples. The gel beads were crushed, and oil droplets were broken up. Next, PCR amplification was performed using cDNA as a template. The products of all GEMs were mixed to construct a standard sequencing library. The cDNAs were clipped into 200-300 bp short sequences. The short sequences were end-repaired, after which poly-A tails, adapter sequences P5 and P7, and sample index sequences were added. Next, the standard sequencing libraries were amplified via PCR. Finally, the libraries were sequenced in high-throughput mode using the PE150 sequencing mode of the Illumina NovaSeq 6000 sequencing platform. After sequencing, the results were annotated against the reference genome, and further bioinformatics analyses were carried out.

### 2.6 SnRNA-seq data processing

Cell Ranger (v3.1.0) was used to perform data quality statistical analysis of the raw data. Reads with low-quality barcodes and UMIs (unique molecular identifiers) were filtered out and then aligned with the reference genome (full-length transcriptomes, as described above). Before quantification, the UMI sequences were corrected for sequencing errors, and valid barcodes were identified based on the EmptyDrops method. The cell-by-gene matrices were produced via UMI counting and cell barcodes calling. Then, the gene matrices for each sample were individually imported into Seurat (v3.1.1) for downstream analysis. Before downstream analysis, we removed doublets with Doublet Finder (v2.0.3). and cells with gene counts of 220-7,300 per cell, UMIs with counts lower than 26,000 per cell were screened for downstream analysis. After retaining high-quality cells, expression was normalized using the common “log homogenization” method. Following data merging with Harmony, PCA was conducted, and the best principal component was selected for subsequent analysis. Based on the previously identified principal components, the Euclidean distance between cells was calculated. Then, Seurat was used to embed cells in an SNN (shared-nearest neighbor) graph based on the Euclidean distance between the cells. Next, the SNN graph was partitioned into highly interconnected quasi-populations according to the Jaccard distance between two cells in local proximity. Finally, the cells were clustered into groups using Louvain’s algorithm, and the data were visualized using a UMAP plot. Subsequently, cell annotation was performed using semi-supervised methods (single R and manual). We used the Seurat rank sum test to filter the genes with upregulated expression in each cell subpopulation. The screening criteria included a gene upregulation fold change (FC) of |log2FC|≥0.36 and p < 0.01, alongside gene expression in more than 25% of cells in the target cluster. The screening criteria for differentially expressed genes between the two groups for each cell type were |log2FC|≥0.36 and p < 0.05 alongside gene expression in more than 10% of cells in the target cluster. We used OmicShare, an online tool based on the R package, to map all peak genes to GO terms or to pathways from the KEGG in the GO database “ http://www.geneontology.org/ (accessed on 8 May 2022)” or KEGG database “https://www.kegg.jp/ (accessed on 8 May 2022)”. The calculated *p* values were corrected via FDR (false discovery rate) to a threshold of FDR ≤ 0.05. The temporal arrangement of cells during PAGA (partition-based graph abstraction) analysis (Scanpy v1.9.1) employed the similarities and dynamic changes in the gene expression patterns to arrange the low-dimensional projection positions of cells and thus reflect the differentiation characteristics between cells. The trajectory analysis was performed using Monocle2 (version 1.0.0).

## 3 Results

### 3.1 SnRNA-seq of purified nucleus from *Decaisnea insignis* fruits

**Figure 1:**
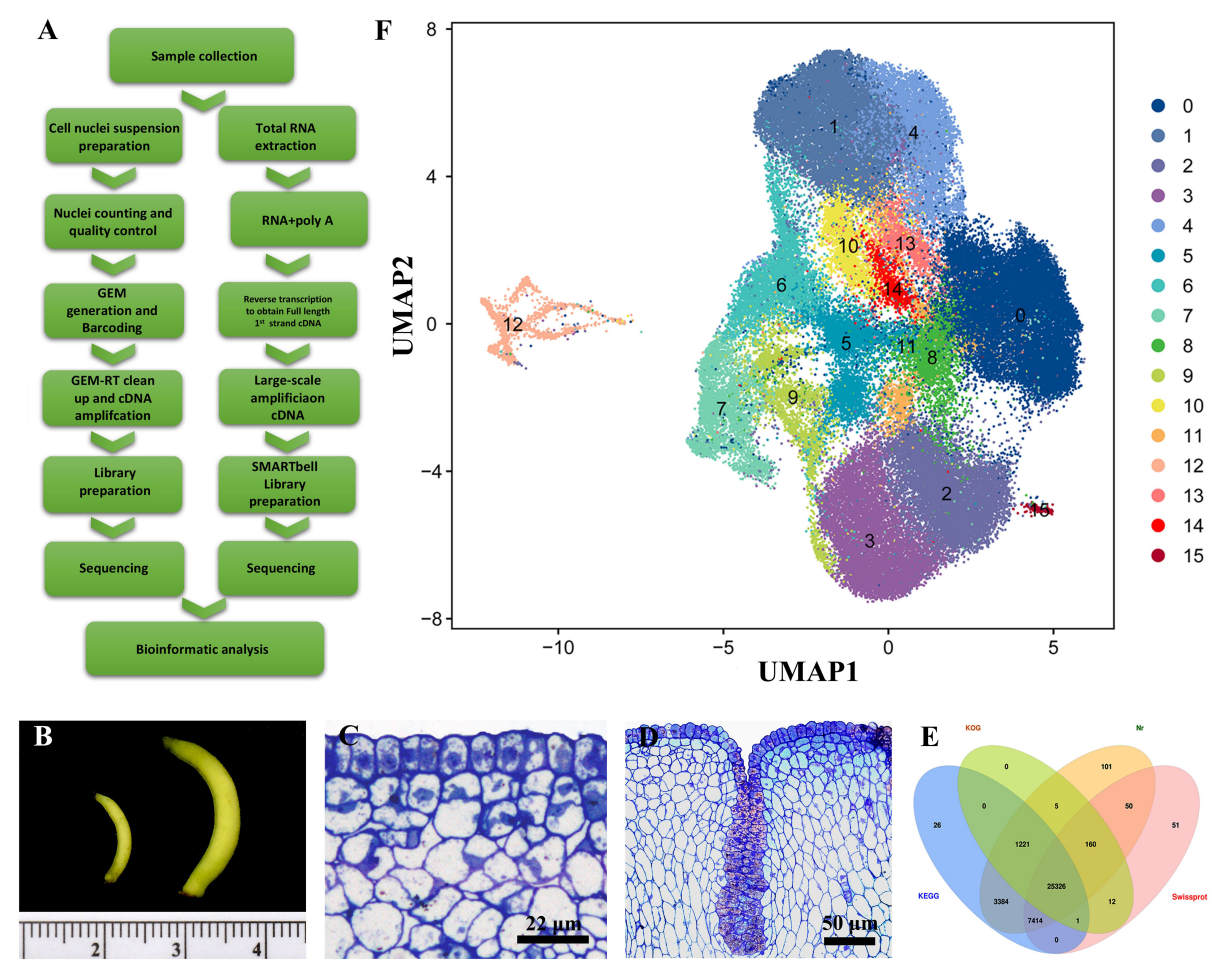
Single nucleus sequencing reveals the development of *Decaisnea insignis* fruits laticiferous canals. **(A)** Workflow for expression analysis of *Decaisnea insignis* fruits by Full-length RNA seq and snRNA-seq. **(B)** *Decaisnea insignis* fruits with diameters of 1 mm (S1 stage) and 2.5 mm (S2 stage) used for extracted snRNA-seq. **(C)** Earliest stage (S1 stage) of the laticiferous canal identified by a layer of epidermal cells with dense cytoplasm and evident nucleus. **(D)** Sunken stage (S2 stage), showing the sunken area formed in the epidermal cells. **(E)** Full-Length RNA-Seq results of mixed fruit samples of 1 mm and 2.5 mm diameter for annotation of snRNA-seq. **(F)** Uniform manifold approximation and projection (UMAP) plot showing cell relationships in a two-dimensional representation. The dimension reduction and cluster identification were performed by Seurat. Dots denote individual cell nucleus (n = 22,260 cells), colors denote different cell clusters (0-15).

To study the cellular characteristics during the developmental progression of *Decaisnea insignis* fruits at a single-cell resolution, we developed a comprehensive workflow (**Figure 1A**) for the snRNA-seq analysis of *Decaisnea insignis* fruits at diameters of 1 mm (S1 stage) and 2.5 mm (S2 stage) (**Figure 1B**). Guided by prior investigations, discernible alterations in the pericarp surface during the early developmental stages of *Decaisnea insignis* fruits were observed. Particularly, through transverse semi-thin sections of *Decaisnea insignis* fruits, we found that the epidermal cells of the fruits were densely arranged during the S1 stage, with no evident differentiation (**Figure 1C**), whereas in the S2 stage, a partial sunken formation was observed on the fruit surface, presenting a noteworthy degeneration phenomenon informed by the dense cytoplasm and expanding nuclei (**Figure 1D**). Motivated by the significance of this observation, we endeavored to elucidate this developmental transition process by employing snRNA-seq technology.

Due to the lack of whole-genome data for *Decaisnea insignis* fruits, we chose to mix a small number of fruit samples and performed the first third-generation long read-length sequencing of *Decaisnea insignis* fruits for full-length transcriptome sequencing to construct the corresponding gene libraries, which were used as the reference genomes. Four databases, KEGG, KOG, Nr and Swissprot, were used to annotate the full-length RNA-seq results, and a total of 25,326 protein-coding genes were annotated (**Figure 1E**).

We then used an established workflow to analyze the snRNA-seq from *Decaisnea insignis* fruits at two different developmental stages, with three biological replicates per stage, to prepare separate snRNA-seq libraries. Each library had at least 385,000,000 reads, with the highest number reaching 482,038,036. The Barcode Q30 for each sample was greater than 97.5%, the RNA read Q30 was greater than 92.8%, and the UMI Q30 was greater than 96.6%. The number of high-quality nuclei in each sample was greater than 13,000, with a maximum of 19,739, and the average number of reads per nucleus was greater than 20,000. After screening, the number of nuclei in each sample was greater than 11,000, up to 16,766. After removing low-quality nuclei, we used Harmony to perform data merging and batch effect correction. Through these steps, we found a total of 16 different cell clusters (0-15). Among them, cluster 0 had the highest number of cell nuclei, reaching 17,828, and cluster 15 had the lowest number of cell nuclei, reaching 166. The results of the cell cluster classification were further visualized using the UMAP (uniform manifold approximation and projection) clustering method (**Figure 1F**).

**Figure 2:**
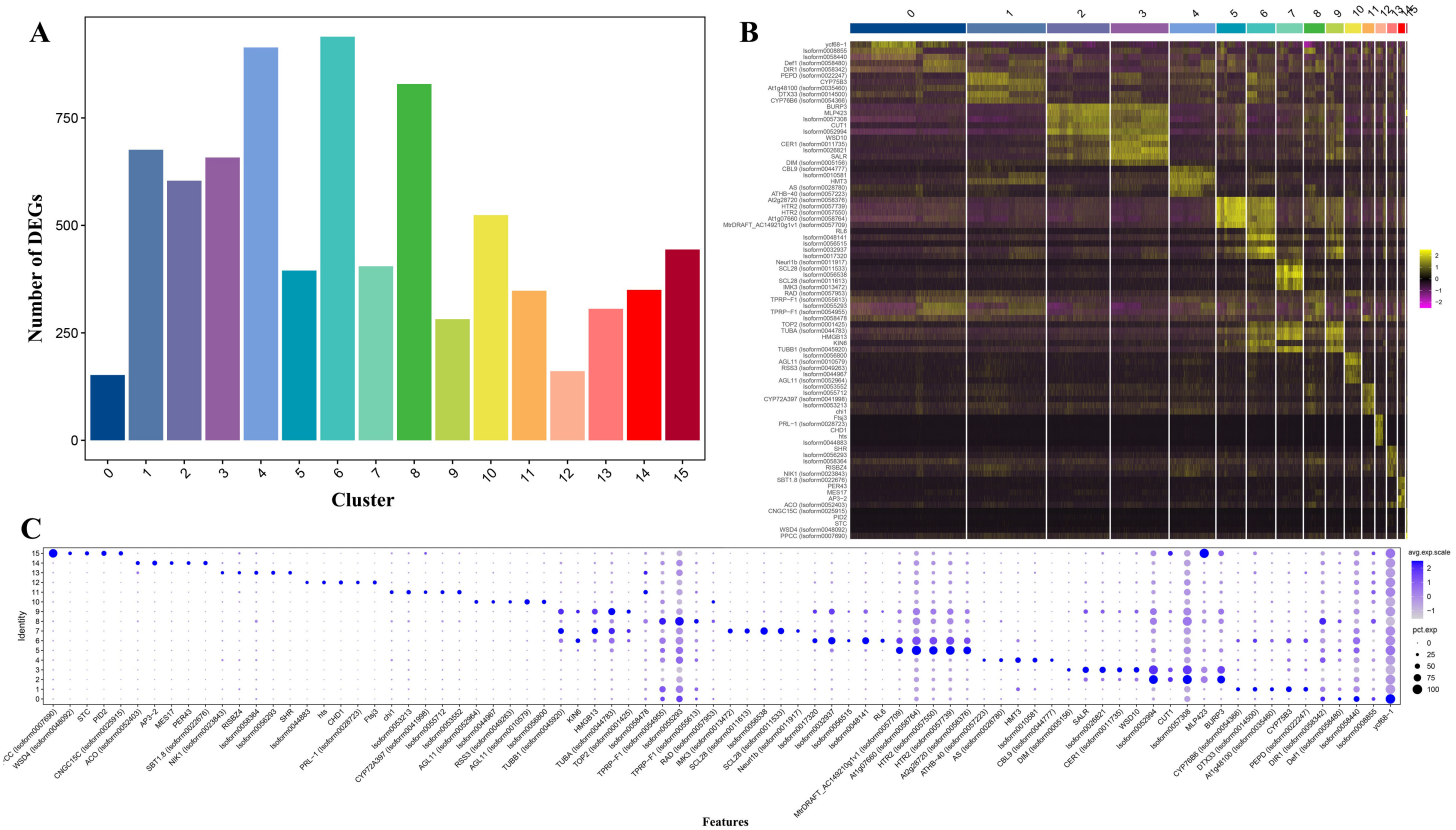
Differentially expressed genes (DEGs) of *Decaisnea insignis* fruits in Single-nucleus sequencing. **(A)** Number of DEGs in all cell clusters. **(B)** Heatmap of DEGs in all cell clusters (Top five up-regulated expressed genes in each cluster). **(C)** Expression of DEGs for all clusters (Top five up-regulated expressed genes in each cluster). The dot diameter indicates the proportion of cluster cells expressing a given gene (ratio), while the color indicates mean expression (exp) across the cells in each cluster.

On this basis, a total of 7,987 up-regulated genes were detected in 16 different clusters, among which the up-regulated genes were more numerous in clusters 4, 6, and 8 than in other clusters. The expressed gene numbers were 914, 939, and 829, respectively (**Figure 2A**). Meanwhile, GO analysis and KEGG analysis were also performed on the up-regulated expressed genes, and the up-regulated expressed genes covered by the 16 clusters were functionally annotated. GO analysis revealed that the GO terms “cellular process” and “metabolic process” from the ontology “Biological Process” had the highest DEGs count among all clusters; two GO terms “catalytic activity” and “binding” from the ontology “Molecular Function” showed the highest DEGs count; and two GO terms “cell part” and “cell” from the ontology “Cellular Component” had the highest DEGs count (**Supplementary Figure S1**). KEGG analysis revealed that the highest number of DEGs was enriched in cluster 8, with a total of 405 DEGs, and the pathways enriched to the most DEGs were “Global and overview maps” and “Translation”. The lowest number of DEGs was enriched in cluster 0, with a total of 65 DEGs, and the pathways “Global and overview maps” and “Translation” were also enriched to the most DEGs (**Supplementary Figure S2**).

On this basis, heat mapping (**Figure 2B**) and bubble mapping (**Figure 2C**) of the genes with better specificity and higher expression levels in different clusters showed that the differentially expressed genes featured a significant degree of differentiation between different clusters, which provided a basis for the subsequent selection of marker genes and other analyses.

### 3.2 *Decaisnea insignis* fruits cell-type annotation of cluster marker genes

**Figure 3:**
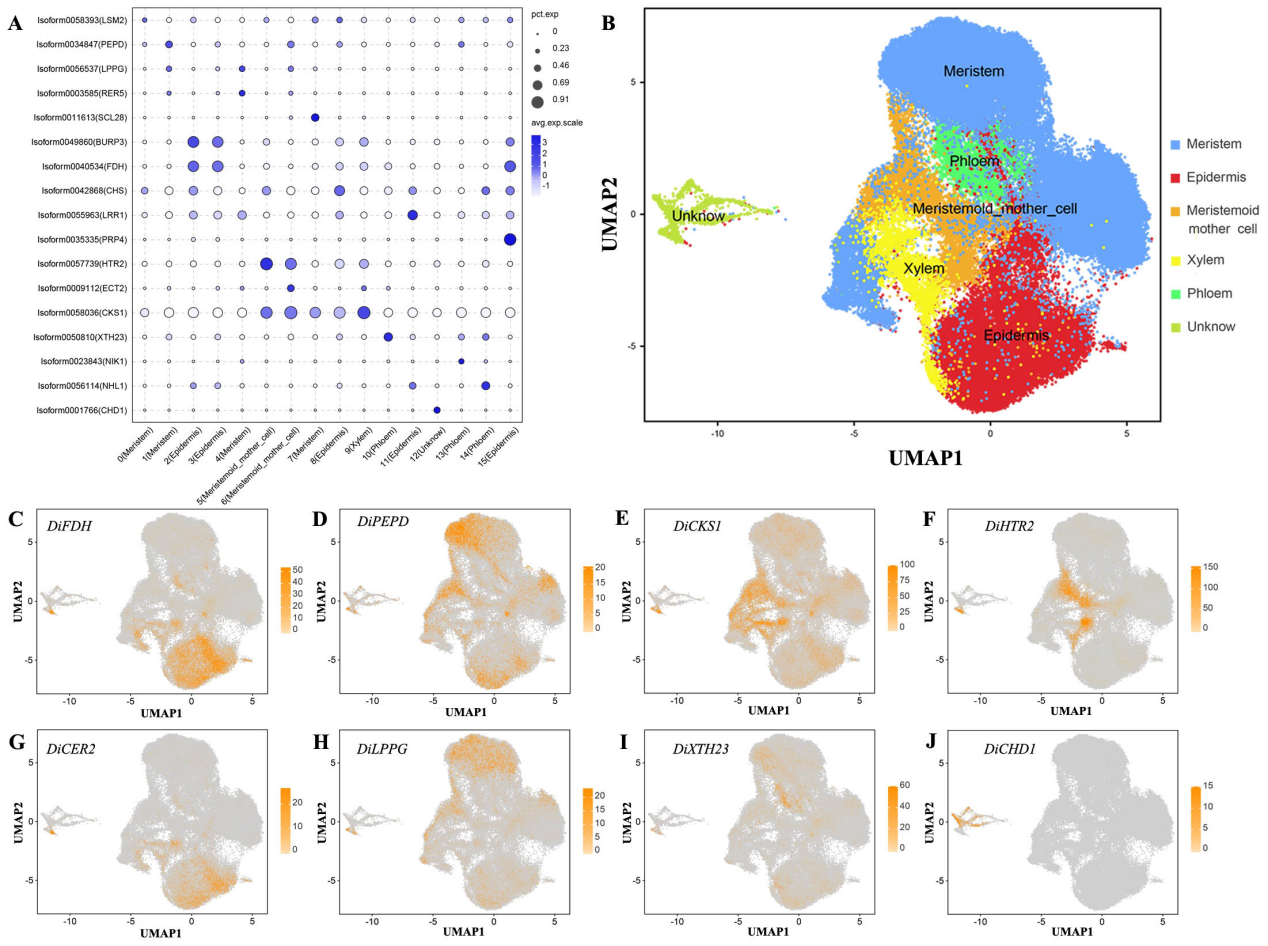
Validate snRNA-seq performance and annotate cell types with known markers. **(A)** Expression of marker gene for all cell types. The dot diameter indicates the proportion of cluster cells expressing a given gene (ratio), while the color indicates mean expression (exp) across the cells in each cluster. **(B)** UMAP visualization after reannotating all cell types. Dots denote individual cell nucleus, and color indicates the cell types. **(C)** UMAP plot of the epidermal cell marker gene *DiFDH.* **(D)** UMAP plot of the meristem cell marker gene *DiPEPD.* **(E)** UMAP plot of the xylem cell marker gene *DiCKS1.* **(F)** UMAP plot of the meristemoid mother cell marker gene *DiHTR2.* **(G)** UMAP plot of the epidermal cell marker gene *DiCER2.* **(H)** UMAP plot of the meristem cell marker gene *DiLPPG.* **(I)** UMAP plot of the phloem cell marker gene *DiXTH23.* **(J)** UMAP plot of the unknow cell marker gene *DiCHD1*. Dots in **(C-J)** denote individual cell nucleus, and color indicates the expression level in a cell.

Based on the functional annotation of up-regulated expressed genes, we used the *Arabidopsis thaliana* cell marker gene to localize the *Decaisnea insignis* fruit cell type. Clusters 2, 3, 8, 11, and 15 were identified as epidermal cells using *FDH* (AT2G26250) and *CER2* (AT4G24510) (Kim et al., 2020); clusters 0, 1, 4, and 7 were identified as meristem cells (**Supplementary Figure S3**) using genes *PEPD* (AT4G29490) and *LPPG* (AT3G50920) (Ryu et al., 2019); clusters 5 and 6 were identified as meristematic mother cells (**Supplementary Figure S4**) using *HTR2* (AT1G09200) (Liu et al., 2020); cluster 9 was identified as xylem cells (**Supplementary Figure S5**) using genes *CKS1* (AT2G27960) (Wendrich et al., 2020); clusters 10, 13, and 14 were identified as phloem cells (**Supplementary Figure S6**) using genes *XTH23* (AT4G25810) (Wendrich et al., 2020); and cluster 12 was identified as unknown cells (**Supplementary Figure S7**) using genes like *CHD1* (AT2G13370). Annotating cell types using marker genes led to the identification of all six previously indicated cells. We created an expression bubble plot (**Figure 3A**) and UMAP plot (**Figure 3B**) depicting the expression levels and number of expressing cells of marker genes. The expression of the chosen marker genes *DiFDH*, *DiCER2*, *DiPEPD*, *DiLPPG*, *DiHTR2*, *DiCKS1*, *DiXTH23*, and *DiCHD1* in *Decaisnea insignis fruits* was clearly very selective and limited to appropriate clusters such as epidermal cells (**Figures 3C, G**), meristem cells (**Figures 3D, H**), meristematic mother cells (**Figure 3F**), xylem cells (**Figure 3E**), phloem cells (**Figure 3I**), and unknown cells (**Figure 3J**), respectively.

### 3.3 Annotation and analysis of differentially expressed genes between clusters of *Decaisnea insignis* fruit epidermal cells

**Figure 4:**
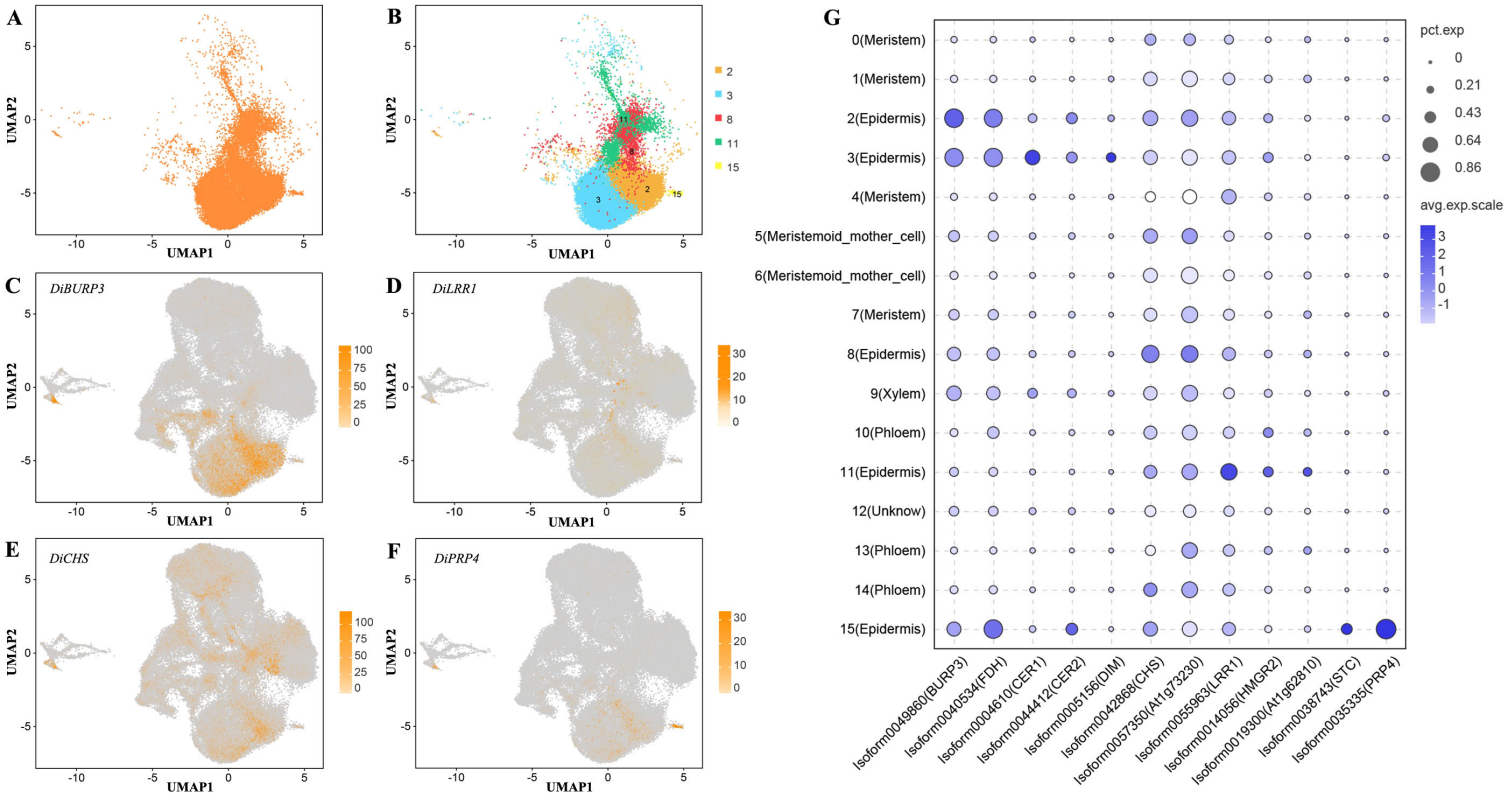
Annotation of *Decaisnea insignis* fruit epidermal cells. **(A)** All epidermal cells UMAP plot. **(B)** UMAP plot of cluster in all constituent epidermal cells. Dots denote individual cell nucleus, and color indicates clusters. **(C)** UMAP plot of the epidermal cell marker gene *DiBURP3.* **(D)** UMAP plot of the epidermal cell marker gene *DiLRR1.* **(E)** UMAP plot of the epidermal cell marker gene *DiCHS.* **(F)** UMAP plot of the epidermal cell marker gene *DiPRP4.* Dots in **(C-F)** denote individual cell nucleus, and color indicates the expression level in a cell. **(G)** Expression of marker genes in all epidermal cells. The dot diameter indicates the proportion of cluster cells expressing a given gene (ratio), while the color indicates mean expression (exp) across the cells in each cluster.

To further deconstruct the biological changes that occur on the pericarp surfaces of Decaisnea insignis fruits in the early stage of development, we conducted a deeper analysis of the epidermal cells (**Figure 4A**). The epidermal cells of Decaisnea insignis fruits consist of five clusters (clusters 2, 3, 8, 11, and 15) (**Figure 4B**), with significant differences in the highly expressed marker genes within the different clusters. DiBURP3 was highly expressed in clusters 2 and 3 (**Figures 4C, G**), DiCHS was highly expressed in cluster 8 (**Figures 4D, G**), DiLRR1 was highly expressed in cluster 11 (**Figures 4E, G**), and DiPRP4 was highly expressed in cluster 15 (**Figures 4F, G**).

**Figure 5:**
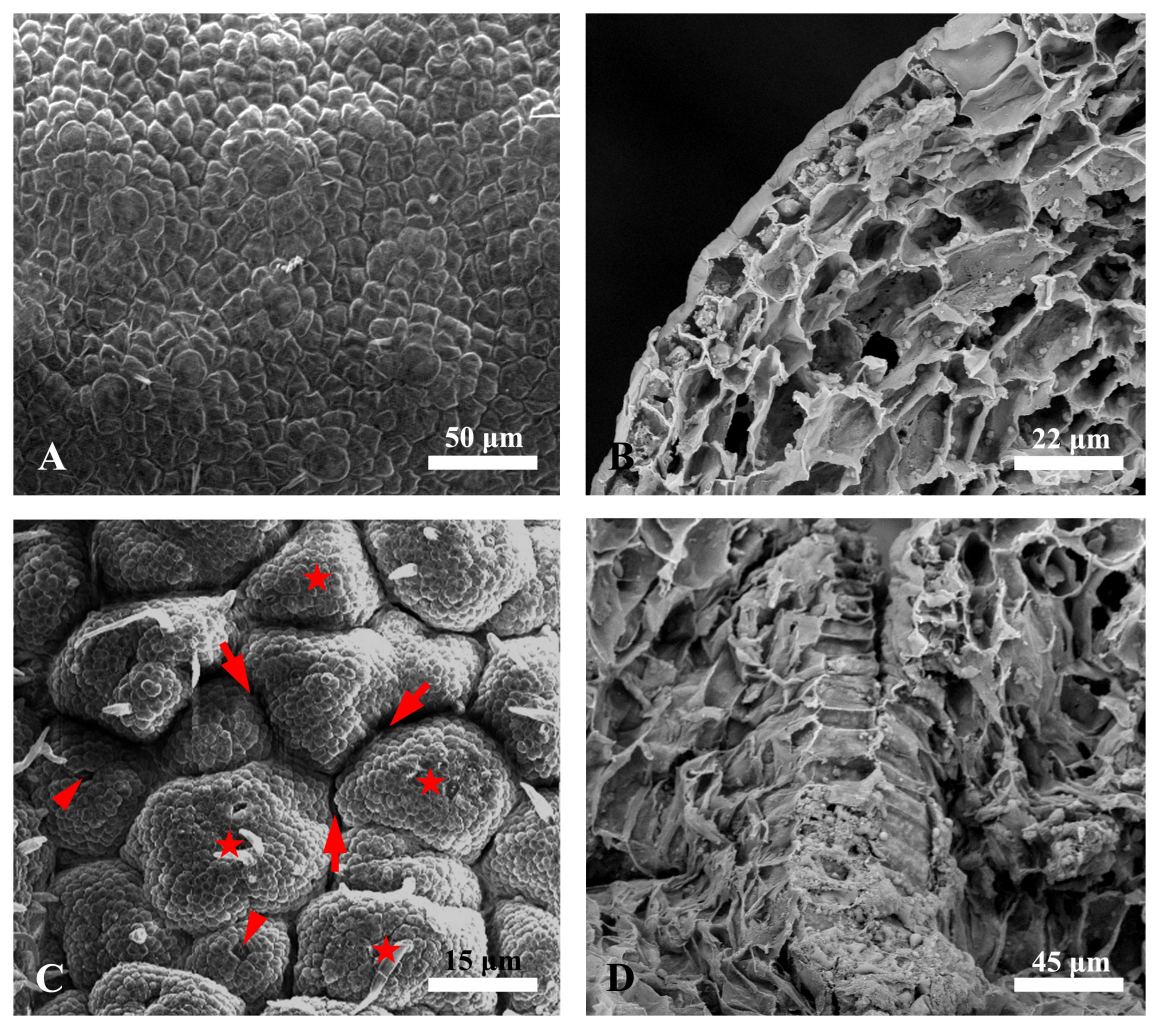
Scanning electron micrographs of *Decaisnea insignis* fruit laticiferous canal. **(A)** Earliest stage, showing the epidermal cells with relatively smooth with no obvious differentiation. **(B)** Cross-section of the earliest epidermis. **(C)** Sunken stage, showing reticulated sunken regions (arrows) enclosing the papillae (asterisks) with stomata (arrowheads) on the top region. **(D)** Cross-section of sunken region.

Then, we further analyzed the ultrastructure of the epidermis of *Decaisnea insignis* fruits via scanning electron microscopy. The results showed that at the earliest stage (S1 stage), the epidermal cells of the ovaries were relatively smooth with no obvious differentiation (**Figures 5A, B**). With the development of the fruit, numerous locations surrounding the stomata of the epidermis simultaneously became activity centers. The cells in the activity center gradually divided and enlarged, forming numerous minute papillae, while the basal regions of the papillae showed reticulated sunken regions that eventually enclosed these papillae. These reticulated sunken regions subsequently formed laticiferous canals (**Figures 5C, D**).

**Figure 6:**
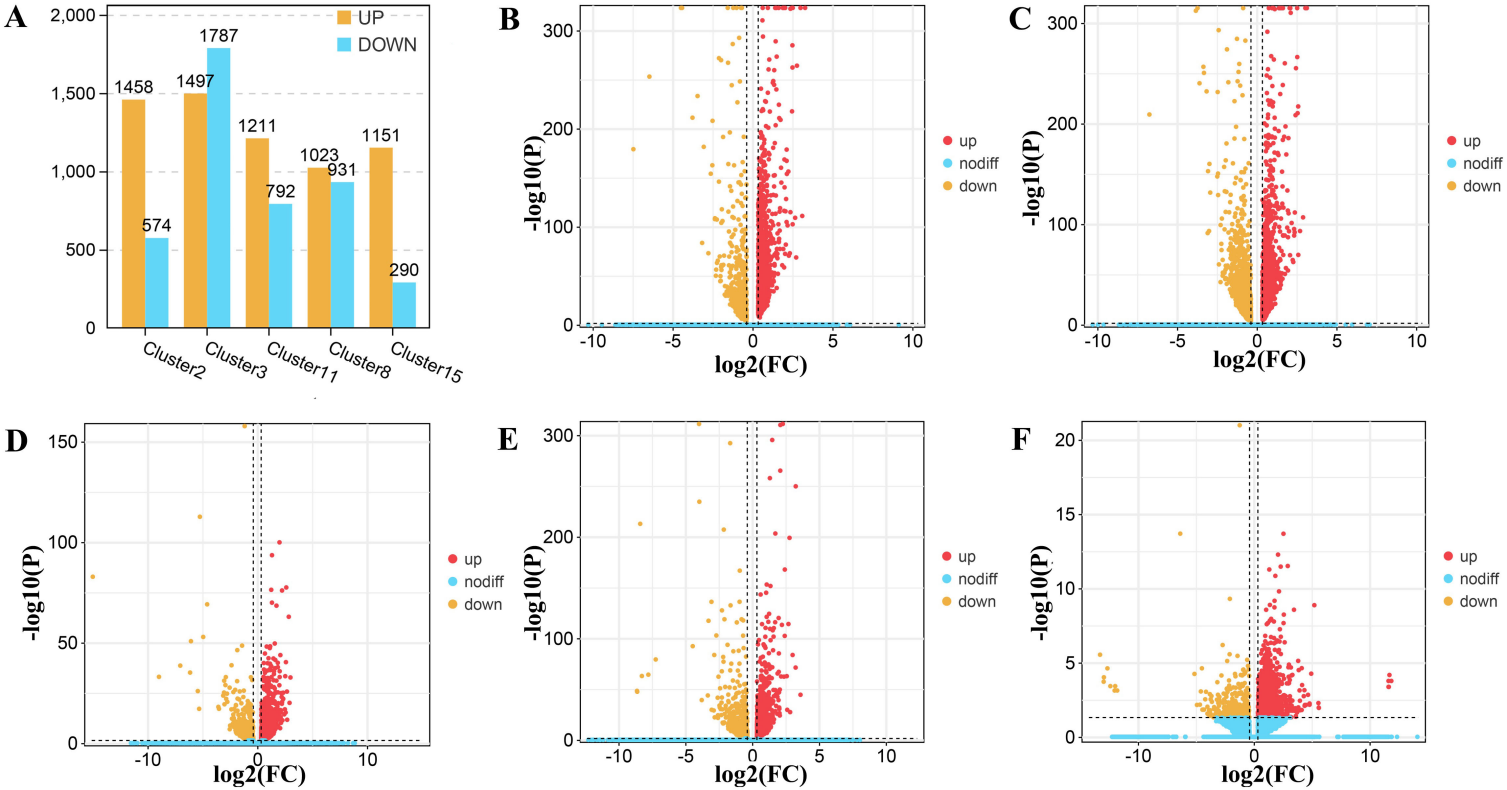
DEGs (differentially expressed genes) in epidermal cells of *Decaisnea insignis* fruit by snRNA-seq. **(A)** DEGs counts in all epidermal cell clusters between two different developmental stages. **(B)** DEGs in different developmental stages within cell cluster 2. **(C)** DEGs in different developmental stages within cell cluster 3. **(D)** DEGs in different developmental stages within cell cluster 11. **(E)** DEGs in different developmental stages within cell cluster 8. **(F)** DEGs in different developmental stages within cell cluster 15. All dots denote individual genes, and color indicates up-regulation or down-regulation of expression levels.

In order to clarify the differences within the epidermal cells and to find the cell clusters in which these changes occurred, we performed a developmental stage (S1 stage vs. S2 stage) difference analysis within the epidermis of *Decaisnea insignis* (**Figure 6**). In the epidermal cell, cluster 2 contained 1,458 up-regulated genes and 574 down-regulated genes, with 285 genes that were uniquely differentially expressed, accounting for 14.02% of all differentially expressed genes (**Figures 6A, B**). Cluster 3 contained 1,497 up-regulated genes, 1,787 down-regulated genes, and 1,198 unique differentially expressed genes, accounting for 36.47% of all differentially expressed genes (**Figures 6A, C**). Cluster 11 contained 1,211 up-regulated genes, 792 down-regulated genes, and 391 unique differentially expressed genes, accounting for 19.52% of all differentially expressed genes (**Figures 6A, D**). We found 1023 up-regulated genes, 931 down-regulated genes, and 466 unique differentially expressed genes in cluster 8, accounting for 23.84% of all differentially expressed genes (**Figures 6A, E**). In cluster 15, we observed 1151 up-regulated genes, 456 down-regulated genes, and 456 unique differentially expressed genes, accounting for 28.37% of all differentially expressed genes (**Figures 6A, F**). In addition, a total of 245 differentially expressed genes overlapped in each cluster (**Supplementary Figure S8**). Through these unique differentially expressed genes, we further analyzed the relationship between the clusters and their functions.

### 3.4 Trajectory analysis of *Decaisnea insignis* fruit epidermal cells to determine the developmental order

**Figure 7:**
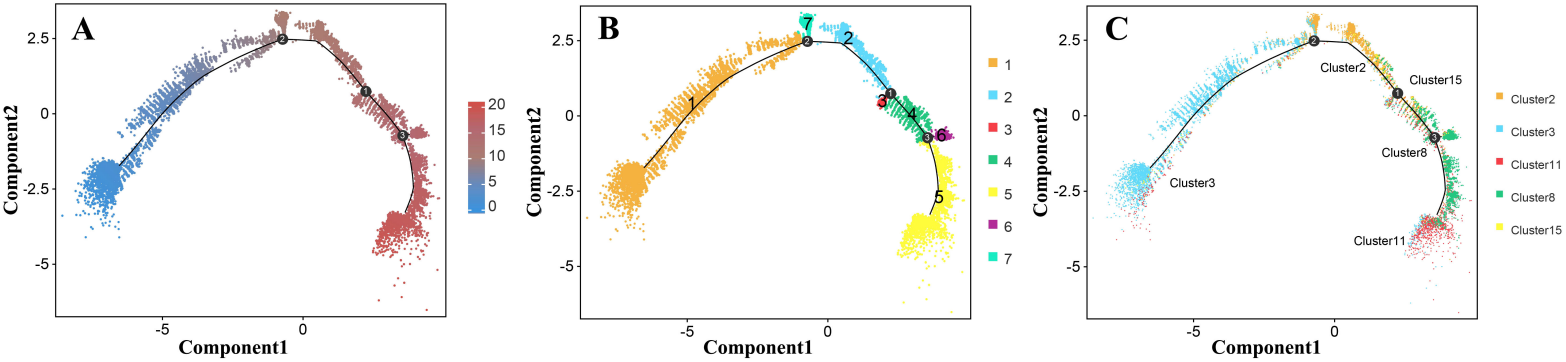
Trajectory analysis of the *Decaisnea insignis* fruit epidermal cells by Monocle2. **(A)** Pseudotime cell trajectory of epidermal cell, colors represent the pseudotime. **(B)** Differentiation state information of epidermal cells, dots denote individual cells and colors denote the state affiliation. **(C)** Cluster information trajectory of epidermal cell visualization, dots denote individual cells and colors denote the epidermal cell cluster affiliation. All the curves indicate pseudotime trajectory.

We also performed a trajectory analysis of the epidermal cells to determine the order of differentiation in the epidermis of *Decaisnea insignis* fruit. The pseudotime trajectories of epidermal cell development were analyzed using Monocle 2 software. Pseudotime values were calculated for all 23,682 epidermal cells, from which the pseudotime trajectories of epidermal cell development were constructed (**Figure 7A**). These trajectories were aggregated into seven different states (1-7) and mapped back to the original five clusters (clusters 2, 3, 8, 11, and 15), where state 1 was mapped to cluster 3, states 7 and 2 were mapped to cluster 2, state 3 was mapped to cluster 15, states 4 and 6 were mapped to cluster 8, and state 5 was mapped to cluster 11 (**Figures 7B, C**). Using state 1 (cluster 3) as the developmental starting point combined with the developmental trajectory, we obtained the differentiation process of epidermal cells. Among all epidermal cells, those in cluster 3 were the first to proliferate. These cells then differentiated into clusters 2 and 15, and a portion of them developed into cluster 8. In addition, some cells in cluster 8 eventually developed into cluster 11. Using a heatmap, the pseudotime of differentially expressed genes was depicted between different states (**Supplementary Figure S9**). In particular, we found that RD21A (**Supplementary Figure S10**), a PCD-related hydrolase, was highly expressed in state 5 (cluster 11), whereas LSD1 (**Supplementary Figure S11**), a negatively regulated PCD transcription factor, was highly expressed in state 6 (cluster 8).

### 3.5 PCD-related genes in *Decaisnea insignis* fruit epidermal cells expressed in specific clusters

According to our previous study, the development of the laticiferous canal in *Decaisnea insignis* fruit is a typical PCD phenomenon, especially from the sunken stage (S2 stage) (Zhou and Liu, 2011). The GO analysis results were applied to look for the genes that might be connected to PCD. The results showed that there were 4080 genes having hydrolase activity (GO: 0140096), of which 108 were identified to be lysosomally located (GO: 0000323). Then, four genes *DiRNA1*, *DiRD21A*, *DiAMC4*, and *DiALEU* exhibiting cysteine-type endopeptidase activity (GO: 0004197) were thought to be possibly associated with PCD. Finally, *DiRD21A* was further analyzed due to the fact that the expression levels of this gene differed the most between S1 stage and S2 stage. Meanwhile, a negatively regulated PCD transcription factor *DiLSD1* was selected for analysis.

**Figure 8:**
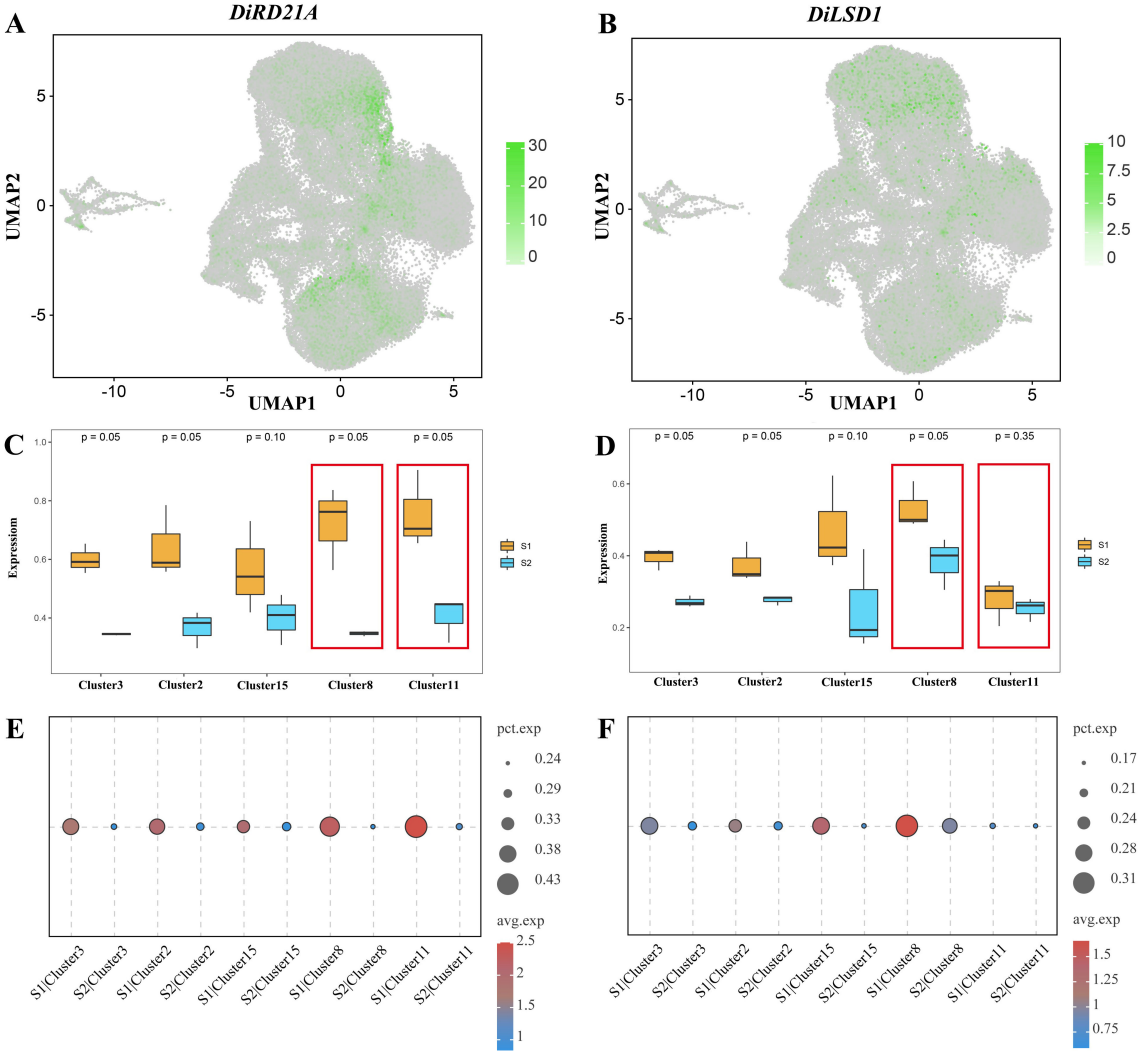
Expression of PCD-related genes in *Decaisnea insignis* fruit epidermis. **(A)** UMAP visualization of expression profiles of *DiRD21A*. **(B)** UMAP visualization of expression profiles of *DiLSD1*. Dots in **(A, B)** denote individual cell nucleus, and color indicates the expression level in a cell. **(C)** Expression of *DiRD21A* in epidermal cell between two different developmental stages. **(D)** Expression of *DiLSD1* in epidermal cell between two different developmental stages. Each box in **(C, D)** indicates maximum value, upper quartile value, median value, lower quartile value and minimum value of the expression levels. **(E)** *DiRD21A* expression bubbles plot at different developmental stages. **(F)** *DiLSD1* expression bubbles plot at different developmental stages. The dot diameter in **(E, F)** indicates the proportion of cluster cells expressing a given gene (ratio), while the color indicates mean expression (exp) across cells in that cluster.

The expression patterns of these two PCD-related genes were different. The *DiRD21A* gene was expressed in several clusters, with a higher number of cells expressed in clusters 8 and 11 than in the other clusters, and higher expression levels in cluster 11 than in cluster 8. In contrast, *DiLSD1* was rarely expressed in the cells of cluster 11. However, we observed a high level of expression in cluster 8 and in the other clusters (**Figures 8A, B**). The results of box plots more intuitively present the expression levels of *DiRD21A* and *DiLSD1* in various clusters of the epidermis. Overall, it can be seen that the expression level of the hydrolase *DiRD21A* gene was higher in the S1 stage than in the S2 stage (p < 0.1). Meanwhile, in the S1 stage, the expression levels of *DiRD21A* in cluster 8 (maximum: 0.8366, upper quartile: 0.7995, median: 0.7623, lower quartile: 0.6631, minimum: 0.5638) and cluster 11 (maximum: 0.9050, upper quartile: 0.8048, median: 0.7046, lower quartile: 0.6800, minimum: 0.6554) were much higher than the averages of these expression levels in other epidermal cells (maximum: 0.7230, upper quartile: 0.6483, median: 0.5737, lower quartile: 0.5420, minimum: 0.5103). Correspondingly, despite showing a trend of higher S1 expression levels than S2, the expression level results for the negatively regulated transcription factor *DiLSD1* in cluster 11 were 0.3293 (maximum), 0.3157 (upper quartile), 0.3021 (median), 0.2532 (lower quartile), and 0.2043 (minimum). This result was much lower than the mean of the expression in other epidermal cell clusters (maximum: 0.5212, upper quartile: 0.4705, median: 0.4199, lower quartile: 0.4051, minimum: 0.3903), with no significant difference between the S1 stage and S2 stage (p > 0.05), (**Figures 8C, D**). Meanwhile, the bubble plots from the analysis of developmental stage differences for the corresponding genes showed consistent results (**Figures 8E, F**).

## 4 Discussion

### 4.1 Construction of snRNA-seq mapping of *Decaisnea insignis* fruit development

In this study, we used a combination of full-length RNA-Seq and snRNA-seq for the first time to analyze and deconstruct the fruit of *Decaisnea insignis*, whose development process currently lacks a reference genome, from molecular and cellular perspectives. We identified a large number of protein-coding genes and classified and identified the major cell types contained in the fruit. About 25,000 protein-coding genes were successfully identified in *Decaisnea insignis* fruits through a library analysis of three biological replicates from two different developmental stages, and six cell types were annotated based on the Arabidopsis marker genes, including the meristem (Ryu et al., 2019; Shulse et al., 2019), epidermis (Kim et al., 2020), phloem (Efroni et al., 2015; Jean-Baptiste et al., 2019; Wendrich et al., 2020), xylem (Wendrich et al., 2020), meristematic mother cells (Liu et al., 2020), and some unknown cells.

According to the structural and developmental analysis, fruit cells involve structures such as the epidermis, phloem, xylem, phloem, and embryo. After fertilization by the male and female gametophytes during the process of fruit development, one or more carpels of the pistil gradually expand, and the cells inside the carpels gradually differentiate into vascular bundles containing xylem and phloem, as well as a large amount of meristem. Then, the seeds in the fruit begin to develop, forming a typical fruit organ (Herrera-Ubaldo and Folter, 2022). This process was also verified more accurately in the present study, in which the epidermis, phloem, and vascular bundles (xylem and phloem), which differentiate earlier and constitute the main part of the fruit, were clearly distinguished and identified, while the embryo, which was not fully developed at the early stage of development, had a smaller number of cells, did not form typical cell taxa, and was not identified. In addition, quantitative analysis of the snRNA-seq UMAP plots showed that epidermis and meristem cells were the most abundant, followed by phloem and xylem cells, while meristem mother cells and unknown cells were less abundant. This result was basically consistent with the conclusions in a previous study on the microstructures in *Decaisnea insignis* fruit development (Hu, 1963).

### 4.2 Expression patterns of PCD-related genes in the developing epidermis of *Decaisnea insignis* fruits

The epidermis, a barrier between the plant and the outside environment, is involved in many biological processes, including transpiration, water and nutrient absorption, and resistance to pathogen invasion (Yang and Ye, 2013; Kundu et al., 2018). Through the accurate identification of target cells, we identified epidermal cell subsets including clusters 2, 3, 8, 11, and 15, with obvious distinctions between these clusters. According to our previous studies, certain epidermal cells can develop into the laticiferous canals (Zhou and Liu, 2011; Zhou et al., 2023b). Unlike their initial divisions, these mature structures typically undergo late differentiation (Sachs and Novoplansky, 1993; Liu et al., 2021). According to the results of the trajectory analysis, the epidermal cells have three differentiation points during the epidermal development of *Decaisnea insignis* fruit. The earliest stage of differentiation corresponded to cluster 3. Subsequently, clusters 2 and 15 formed independently. Finally, some cells differentiated into clusters 8 and 11. This result suggests that cluster 8 and cluster 11 are most likely the cells that form the secretory structures of laticiferous canals via PCD. Correspondingly, we found that the PCD-related genes *DiRD21A* and *DiLSD1* were uniquely expressed in cluster 8 and cluster 11. In a previous study on *Arabidopsis thaliana*, Yang et al. (2007) found that RD21A exert the function of cysteine proteinase/thiol protease to hydrolyze proteins involved in PCD. Similarly, there are hydrolases such as RD19A, THI1, RD19C that perform the same function (Yang et al., 2007). These hydrolases are regulated by transcription factors such as SPL (sporocyteless/nozzle) and EXS (excess microsporocytes 1/extra sporogenous cells) (Zhang et al., 2014). However, it was showed that LSD1, a transcription factor of zinc finger protein, can negatively regulate red light-induced programmed cell death in Arabidopsis through antagonistic regulation of HY5 (Chai et al., 2015). In brief, *DiRD21A* and *DiLSD1* in the cell clusters 8 and 11 in the epidermis suggests that these cells may be congruent with sunken regions forming the laticiferous canals observed in the ultrastructure.

As two different developmental stages, S1 and S2, group difference comparisons were able to accurately capture the changes in different genes during different developmental stages. Based on the results of the group difference analysis, we found that the expression level of *DiRD21A* hydrolase was higher in cluster 8 and 11 than other clusters. At the same time, the expression level of *DiRD21A* in cluster 11 was higher than that in cluster 8, whereas *DiRD21A* in S1 and S2 showed a pattern of anteriorly high and posteriorly low expression levels respectively, which means that the expression of some PCD-related genes preceded the relevant biological activities. Meanwhile, we found that LSD1, a zinc finger protein transcription factor capable of negatively regulating PCD, was highly expressed only in cluster 8, with lower expression levels in cluster 11. We also observed a pattern showing higher expression levels of S1 and lower expression levels of S2. This result suggests that the PCD phenomenon may have been suppressed in cluster 8, whereas in the cells of cluster 11, which lacks the suppression of LSD1, the PCD phenomenon proceeded normally, leading to the formation of sunken regions (early laticiferous canals). And the results are consistent with the results of second-generation sequencing, as well as the qPCR validation results in a previous study (Zhou et al., 2023b).

Taken together, while other cells in the epidermis of *Decaisnea insignis* fruits develop normally, some cells may experience the PCD process due to the combined action of transcription factors and hydrolases, thus creating a sunken area, which will form complete laticiferous canals as the fruit matures and develops in the near future.

### 4.3 Advantages of combining snRNA-seq with cytoarchitecture in the developmental study of secretory structures

The quest to demystify the role of programmed cell death (PCD) in the genesis of secretory structures, such as laticiferous canals, has been problematized by the methodological limitations inherent in traditional investigations of cell biology. Despite providing insights into morphological changes, conventional histological methods often fall short in capturing the dynamic gene expression profiles that dictate developmental outcomes (Gaffal et al., 2007; Chen and Wu, 2010; Zhou et al., 2012; Gui and Liu, 2014). Likewise, the application of second-generation sequencing techniques, although beneficent in detailing bulk transcriptomic data, lacks the spatial and cellular resolution required to delineate the nuanced, cell-specific transcriptional changes that occur during PCD and the formation of complex secretory tissues (Zhou et al., 2023b). Such techniques are unable to resolve the heterogeneity within mixed cell populations or capture the transient gene expression states that are pivotal during secretory structure ontogeny. This limitation creates a significant gap in our understanding, as the interplay between gene expression and cellular context is crucial in elucidating PCD pathways and their roles in the development of secreting structures. These traditional approaches also have disadvantages in precisely isolating rare cell types, such as those undergoing PCD within laticiferous canals, further obscuring our understanding of these crucial developmental events. Therefore, a more sophisticated strategy is required, one that can simultaneously offer high-throughput genomic data at a single-cell resolution and provide spatial and temporal context to deconstruct the complex molecular mechanisms underlying the development of PCD and secretory structures. The methodological framework in this study is predicated on an integrative approach that harnesses the power of snRNA-seq alongside ultrastructural analysis. snRNA-seq enable the high-resolution dissection of gene expression patterns by isolating individual nuclei from heterogeneous tissue samples, subsequently enabling their transcriptomes to be sequenced. This is particularly advantageous for secretory tissues such as laticiferous canals, which present a complex cellular composition and a dynamic range of developmental stages. By complementing snRNA-seq with an ultrastructural analysis using electron microscopy, one can obtain a holistic view of the cellular architecture and its corresponding transcriptional profiles during key developmental processes. This dual approach not only circumvents the limitations of conventional bulk sequencing methods, which may obscure the contributions of distinct cell types to overall tissue function, but also remains superior to traditional histological methods that lack the resolution to map intricate patterns of gene expression. The synergy between snRNA-seq and ultrastructural assessment embodied in our methodology was strategically designed to provide novel insights into the processes facilitating programmed cell death and, consequently, the intricate development of laticiferous canals in *Decaisnea insignis* fruits.

### 4.4 Relationship between PCD and rubber synthesis in the laticiferous canals of *Decaisnea insignis* fruits

Our previous studies have confirmed that rubber particles in the latex canal of *Decaisnea insignis* are mainly accumulated after the degradation of protoplasts (Zhou and Liu, 2011; Zhou et al., 2023b). At S1 stage (earliest stage), the epidermal cells with no obvious differentiation showed a small amount of brown flocculent or irregular material, i.e., rubber precursors (Zhou et al., 2023b). Cytologically, the PCD characters at this stage had not yet been observed, however in cluster 11 and cluster 8, which identified as cells that will develop into laticiferous canal, PCD-related genes had begun to be expressed. Specifically, the difference in *DiLSD1* expression levels determines the final differentiation of cluster 11 and cluster 8 into the secretory epidermal cells and gap cells, respectively. Cluster 8, which has a higher level of *DiLSD1* expression, has an inhibited PCD process and eventually differentiates into gap cells. In contrast, cluster 11 with lower *DiLSD1* expression levels had normal PCD and differentiated into the secretory epidermal cells. At S2 stage (sunken stage), the secretory epidermal cells in sunken regions (cluster 11) showed an increase in the amount of osmiophilic material, and in contrast, there were little amount of osmiophilic material in gap cells (cluster 8) of the sunken regions (Zhou et al., 2023b). At the same stage, the secretory epidermal cells showed an exhibiting unique early symptom of degeneration in the ultrastructure and TUNEL-positive nuclei reaction, while gap cells of the sunken regions showed a TUNEL-negative reaction (Zhou et al., 2023b). Obviously, both PCD process and rubber synthesis were observed in the secretory epidermal cells (cluster 11) at S2 stage. Our studies suggest rubber synthesis and the PCD process in the laticiferous canal of *Decaisnea insignis* occur in the same time and space, and a strong correlation could be existed between the two biological processes at the ultrastructural and single-cell level.

### 4.5 Roles of PCD in plant secretory structures

PCD is an important cellular physiological process that leads to cellular self-destruction for plant tissue construction and organ development (Lee and Chen, 2002; Kwon et al., 2010; He and Wu, 2013; Huai et al., 2021; Wang et al., 2021). In plant glands, different modes of developmentally regulated PCD have been widely observed in the mucilage cells of *Araucaria angustifoliain* (Mastroberti and Mariath, 2008), floral nectaries of *Digitalis purpurea* (Gaffal et al., 2007) and *Ipomoea purpurea* (Gui and Liu, 2014), *Gossypium hirsutum* pigment glands (Liu et al., 2010; Wang et al., 2016), *Dictamnus dasycarpus* trichome cavities (Zhou et al., 2012) and capitate glandular hairs (Zhou et al., 2023a), laticiferous canal formation of *Decaisnea insignis* (Zhou and Liu, 2011; Zhou et al., 2023b), and secretory cavities in *Citrus sinensis* (Chen and Wu, 2010) and *Dictamnus dasycarpus* (Zhou et al., 2014), and ultimately involved in various plant secretory structure construction, during which process secretions always occur or accumulate after various organelle or protoplast degradation. Collectively, it could be concluded that PCD may play critical roles in the secretory construction and secretion accumulation in plant secretory structures.

## 5 Conclusions

In this study, the snRNA-seq approach was combined with validation via ultrastructure observations and trajectory analysis to offer an integrated method to investigate the cell census of *Decaisnea insignis* fruit, which would otherwise be difficult to examine via scRNA-seq because of the recalcitrance of such cells to protoplasting. The comprehensive *Decaisnea insignis* fruit snRNA-seq cell census in this study enabled us to explore the PCD phenomena pertaining to the developmental trajectory of the laticiferous canal at the single-cell level and provided a step forward to better understand the development of secretory structures and the regulatory networks of interconnected genes.

Overall, our study serves as a foundation for more in-depth studies on the development of secretory structures and internal secondary metabolite synthesis at a single-cell level. Further integration of RNA-seq with other rapidly evolving single-cell genomics technologies will help us comprehensively characterize the cellular properties underlying plant development and material synthesis and provide relevant technological innovations for further probing secondary metabolite synthesis among secretory structures.

## Data Availability

The data presented in the study are deposited in the Genome Sequence Archive in BIG Data Center (https://bigd.big.ac.cn/), Beijing Institute of Genomics (BIG), Chinese Academy of Sciences, under the accession number: CRA016938 (Full-length RNA sequencing) and CRA016989 (Single-nucleus sequencing).

## Supplementary material

Supplementary Table 1 All cell clusters up-regulated gene list (Top50).

Supplementary Table 2 Marker genes annotation list.

Supplementary Figure 1 All cell clusters up-regulated gene GO annotation results. The DEGs were annotated to biological process (BP), molecular function (MF), and cellular component (CC) GO terms in the GO database.

Supplementary Figure 2 All cell clusters up-regulated gene KEGG annotation result The DEGs were mapped to the KEGG database to categorized the enrichment of the DEGs.

Supplementary Figure 3 The bubble plot of meristem cells marker genes. Expression of marker gene for all meristem cells. The dot diameter indicates the proportion of cluster cells expressing a given gene (ratio), while the color indicates mean expression (exp) across cells in each cluster.

Supplementary Figure 4 The bubble plot of meristematic mother cells marker genes. Expression of marker gene for all meristematic mother cells. The dot diameter indicates the proportion of cluster cells expressing a given gene (ratio), while the color indicates mean expression (exp) across the cells in each cluster.

Supplementary Figure 5 The bubble plot of xylem cells marker genes. Expression of marker gene for xylem cells. The dot diameter indicates the proportion of cluster cells expressing a given gene (ratio), while the color indicates mean expression (exp) across the cells in each cluster.

Supplementary Figure 6 The bubble plot of phloem cells marker genes. Expression of marker gene for phloem cells. The dot diameter indicates the proportion of cluster cells expressing a given gene (ratio), while the color indicates mean expression (exp) across the cells in each cluster.

Supplementary Figure 7 The bubble plot of cluster 12 Unknown Cell marker genes. Expression of marker gene for cluster 12 cells. The dot diameter indicates the proportion of cluster cells expressing a given gene (ratio), while the color indicates mean expression (exp) across the cells in each cluster.

Supplementary Figure 8 Venn plot between two different development stages of the DEGs in the epidermis of Decaisnea insignis fruit. DEGs in the epidermis between two developmental stages (S1 stage vs. S2 stage), showing the number of overlap and unique DEGs in each cluster.

Supplementary Figure 9 The DEGs of pseudotime between different states. Gene expression heatmap for all states of epidermal cells.

Supplementary Figure 10 Scatter plot of the expression level of PCD-related gene *DiRD21A* at different states on the pseudotime line. Showing the trend of the DiRD21A expression along pseudotime trajectory during differentiation of the epidermal cells. A dot in the scatterplot represents a single nucleus. The curve is a trend line. The X-axis was defined as “Pseudotime” and Y-axis was defined as “Relative expression” by log10 (exp + 1).

Supplementary Figure 11 Scatter plot of the expression level of PCD-related gene *DiLSD1* at different states on the pseudotime line. Showing the trend of the DiLSD1 expression along pseudotime trajectory during differentiation of the epidermal cells. A dot in the scatterplot represents a single nucleus. The curve is a trend line. The X-axis was defined as “Pseudotime” and Y-axis was defined as “Relative expression” by log10 (exp + 1).
