# Supplementary figures and images for: Full-length RNA sequencing and single-nucleus sequencing deciphers programmed cell death and developmental trajectories in laticiferous canals of Decaisnea insignis fruits
Source: Front Plant Sci. 2024 Aug 13;15:1446561. doi: 10.3389/fpls.2024.1446561 (PMC11369900; doi:10.3389/fpls.2024.1446561)

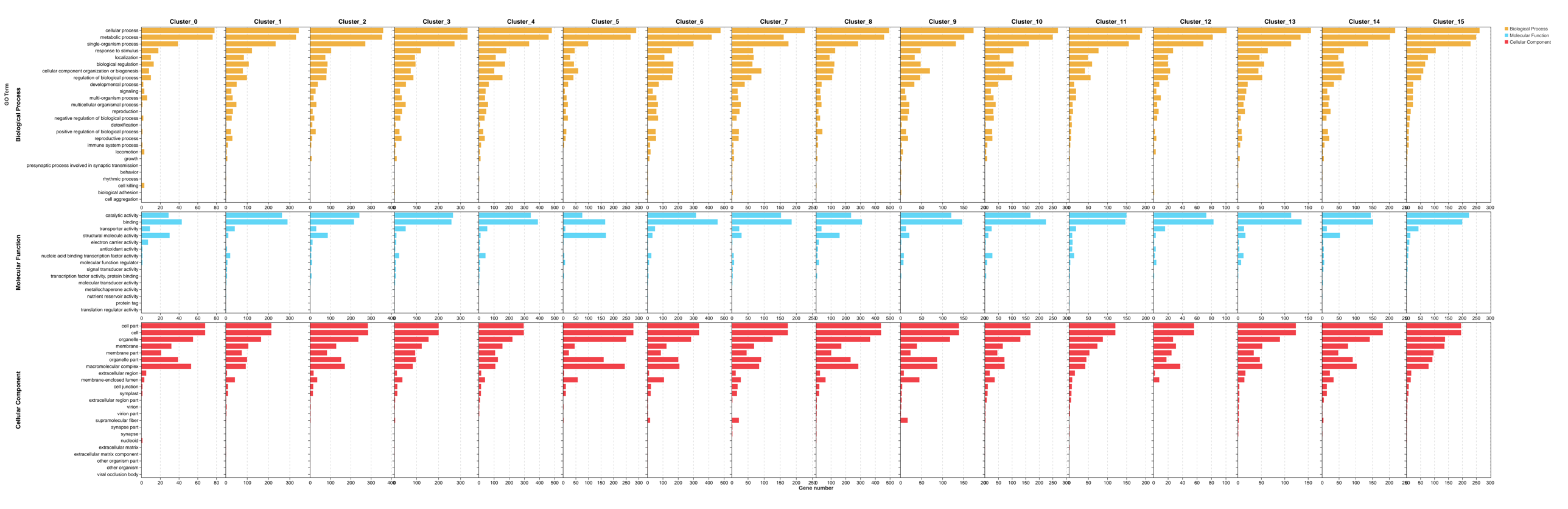

Supplement: Supplementary Figure 1 — All cell clusters up-regulated gene GO annotation results. The DEGs were annotated to biological process (BP), molecular function (MF), and cellular component (CC) GO terms in the GO database. [file Image_1.pdf]

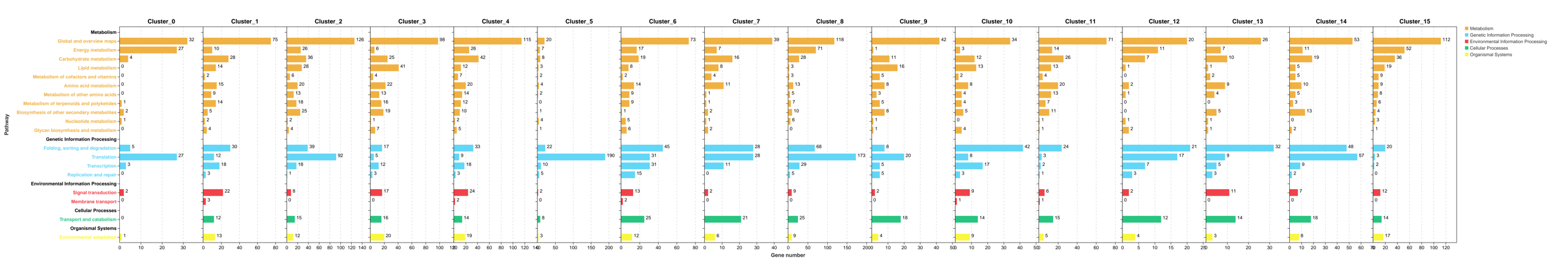

Supplement: Supplementary Figure 2 — All cell clusters up-regulated gene KEGG annotation result The DEGs were mapped to the KEGG database to categorized the enrichment of the DEGs. [file Image_2.pdf]

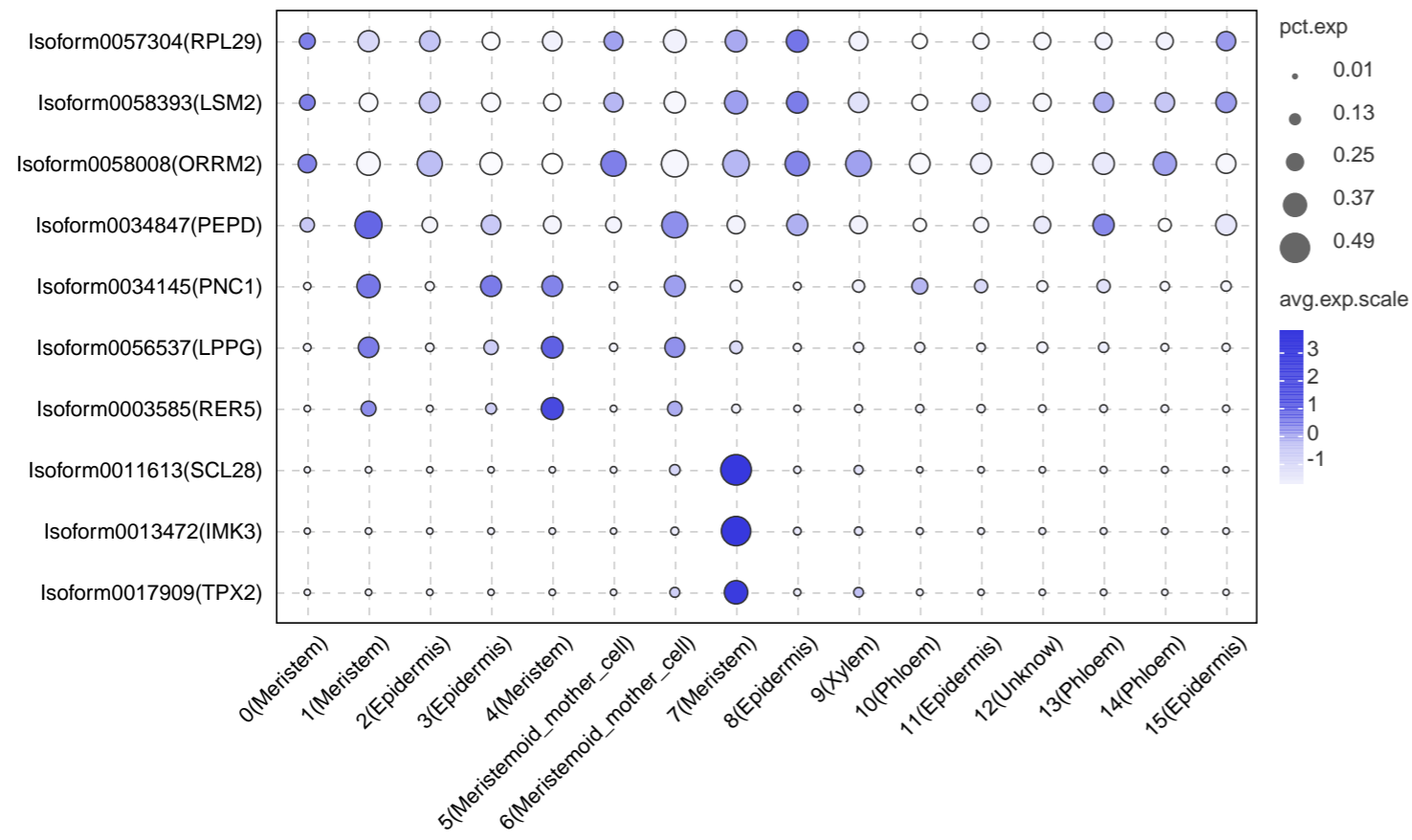

Supplement: Supplementary Figure 3 — The bubble plot of meristem cells marker genes. Expression of marker gene for all meristem cells. The dot diameter indicates the proportion of cluster cells expressing a given gene (ratio), while the color indicates mean expression (exp) across cells in each cluster. [file Image_3.pdf]

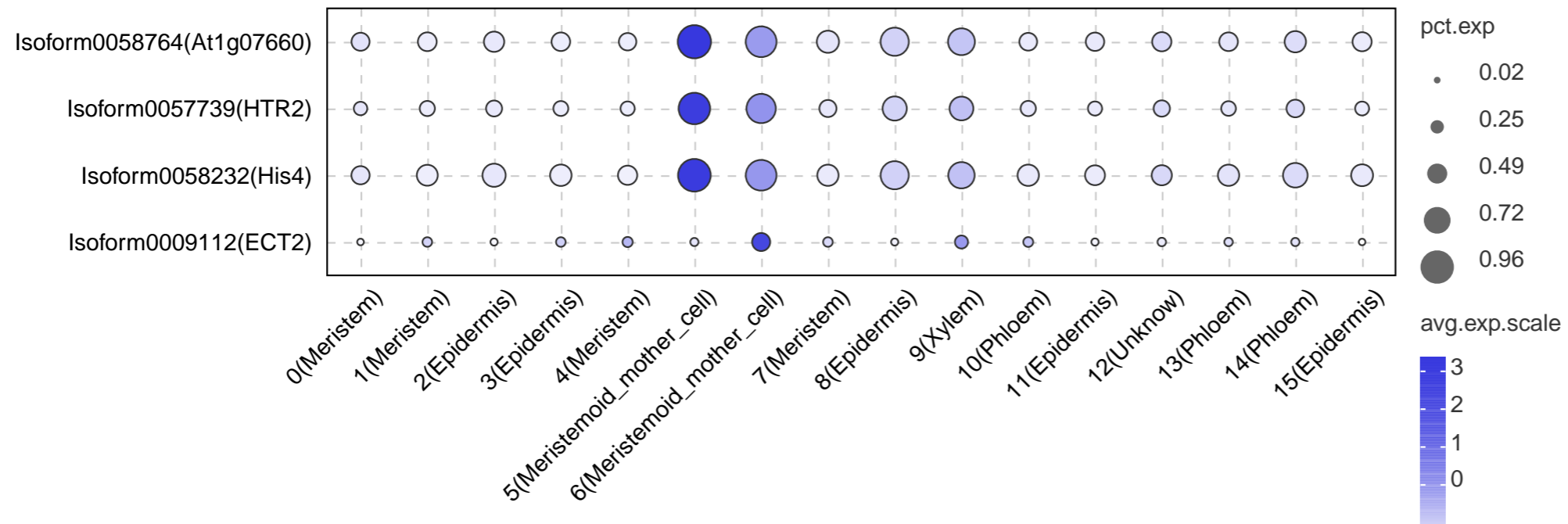

Supplement: Supplementary Figure 4 — The bubble plot of meristematic mother cells marker genes. Expression of marker gene for all meristematic mother cells. The dot diameter indicates the proportion of cluster cells expressing a given gene (ratio), while the color indicates mean expression (exp) across the cells in each cluster. [file Image_4.pdf]

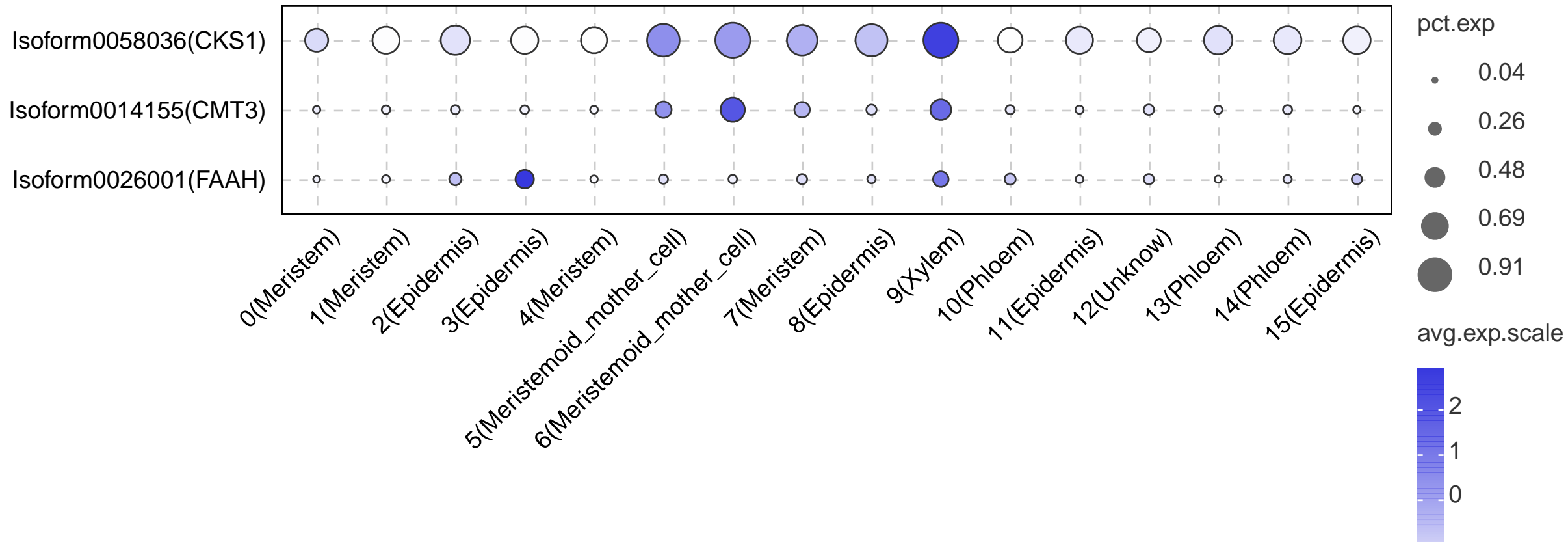

Supplement: Supplementary Figure 5 — The bubble plot of xylem cells marker genes. Expression of marker gene for xylem cells. The dot diameter indicates the proportion of cluster cells expressing a given gene (ratio), while the color indicates mean expression (exp) across the cells in each cluster. [file Image_5.pdf]

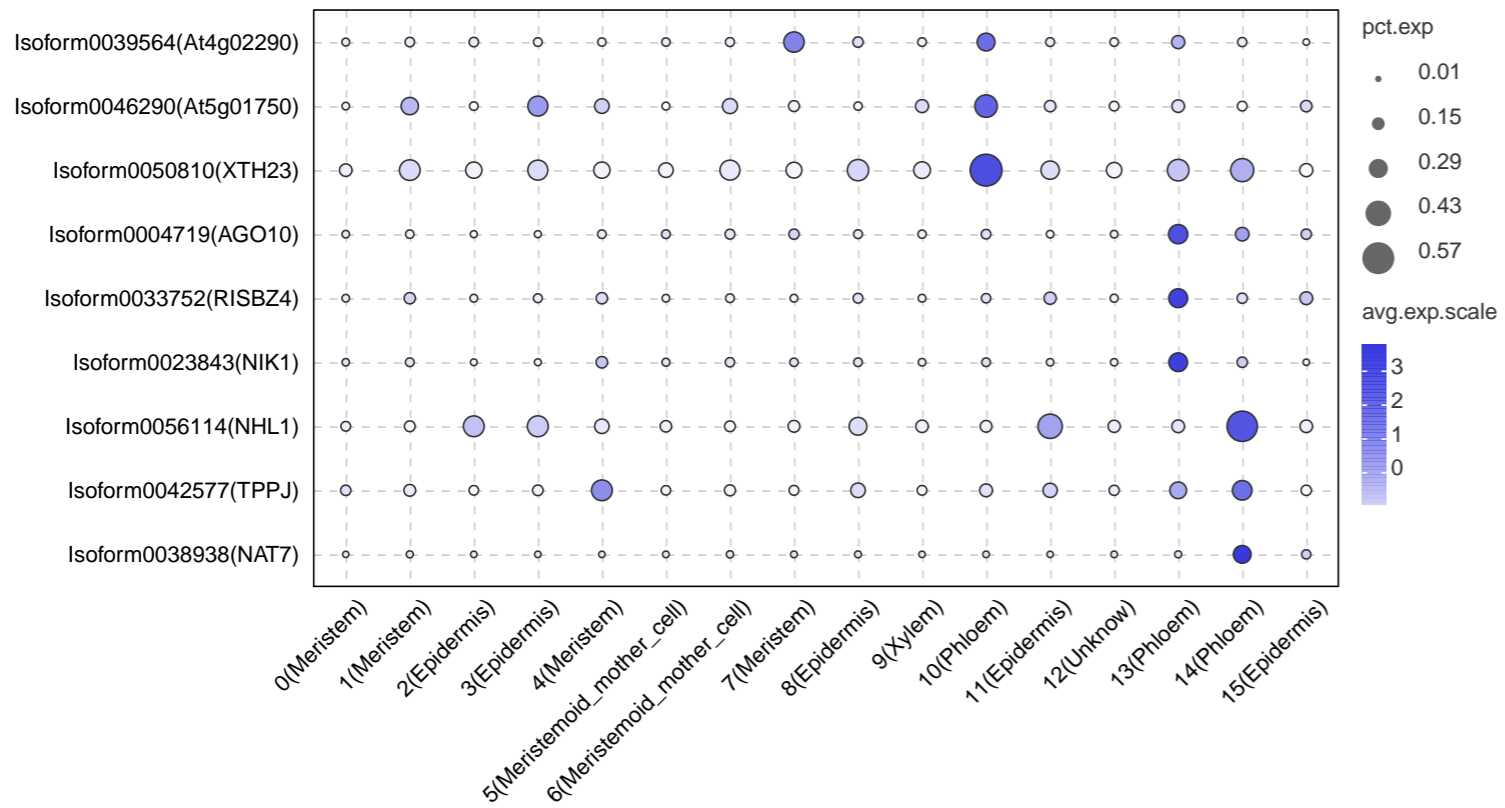

Supplement: Supplementary Figure 6 — The bubble plot of phloem cells marker genes. Expression of marker gene for phloem cells. The dot diameter indicates the proportion of cluster cells expressing a given gene (ratio), while the color indicates mean expression (exp) across the cells in each cluster. [file Image_6.pdf]

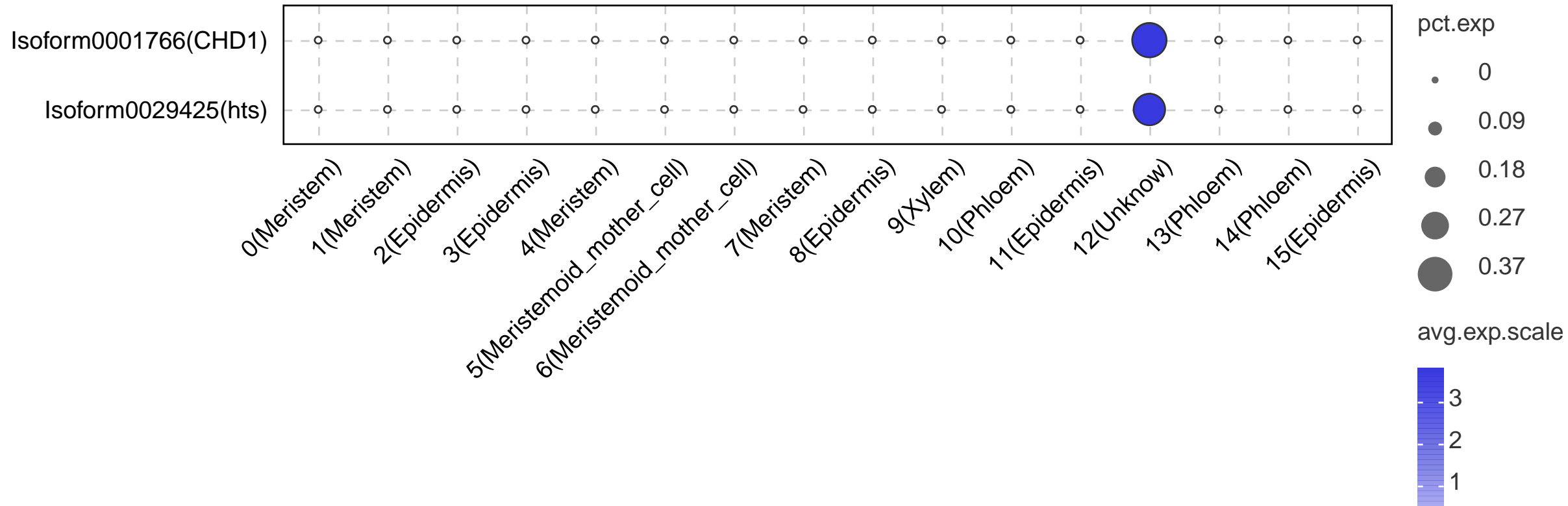

Supplement: Supplementary Figure 7 — The bubble plot of cluster 12 Unknown Cell marker genes. Expression of marker gene for cluster 12 cells. The dot diameter indicates the proportion of cluster cells expressing a given gene (ratio), while the color indicates mean expression (exp) across the cells in each cluster. [file Image_7.pdf]

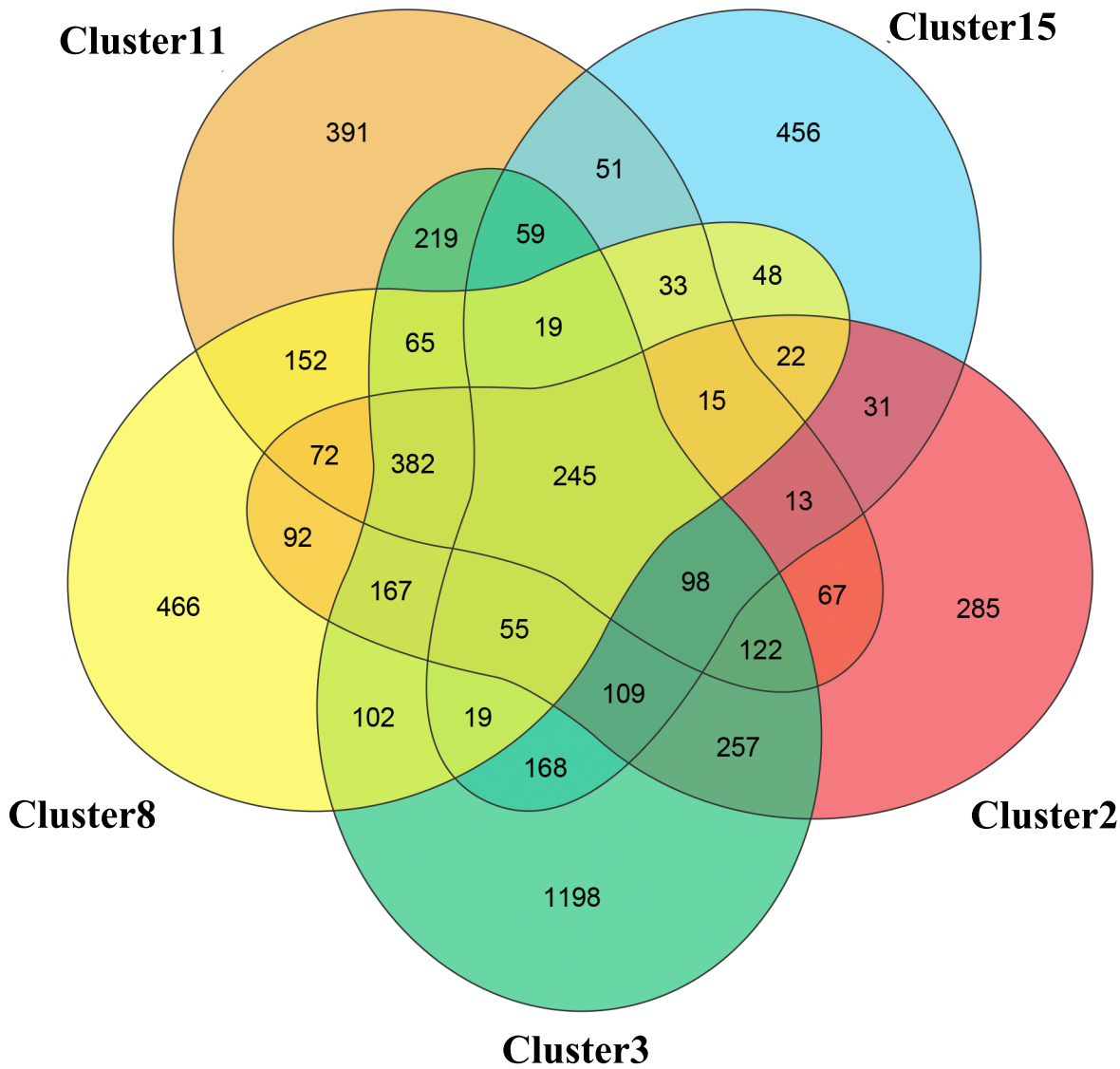

Supplement: Supplementary Figure 8 — Venn plot between two different development stages of the DEGs in the epidermis of Decaisnea insignis fruit. DEGs in the epidermis between two developmental stages (S1 stage vs. S2 stage), showing the number of overlap and unique DEGs in each cluster. [file Image_8.pdf]

# DiRD12A

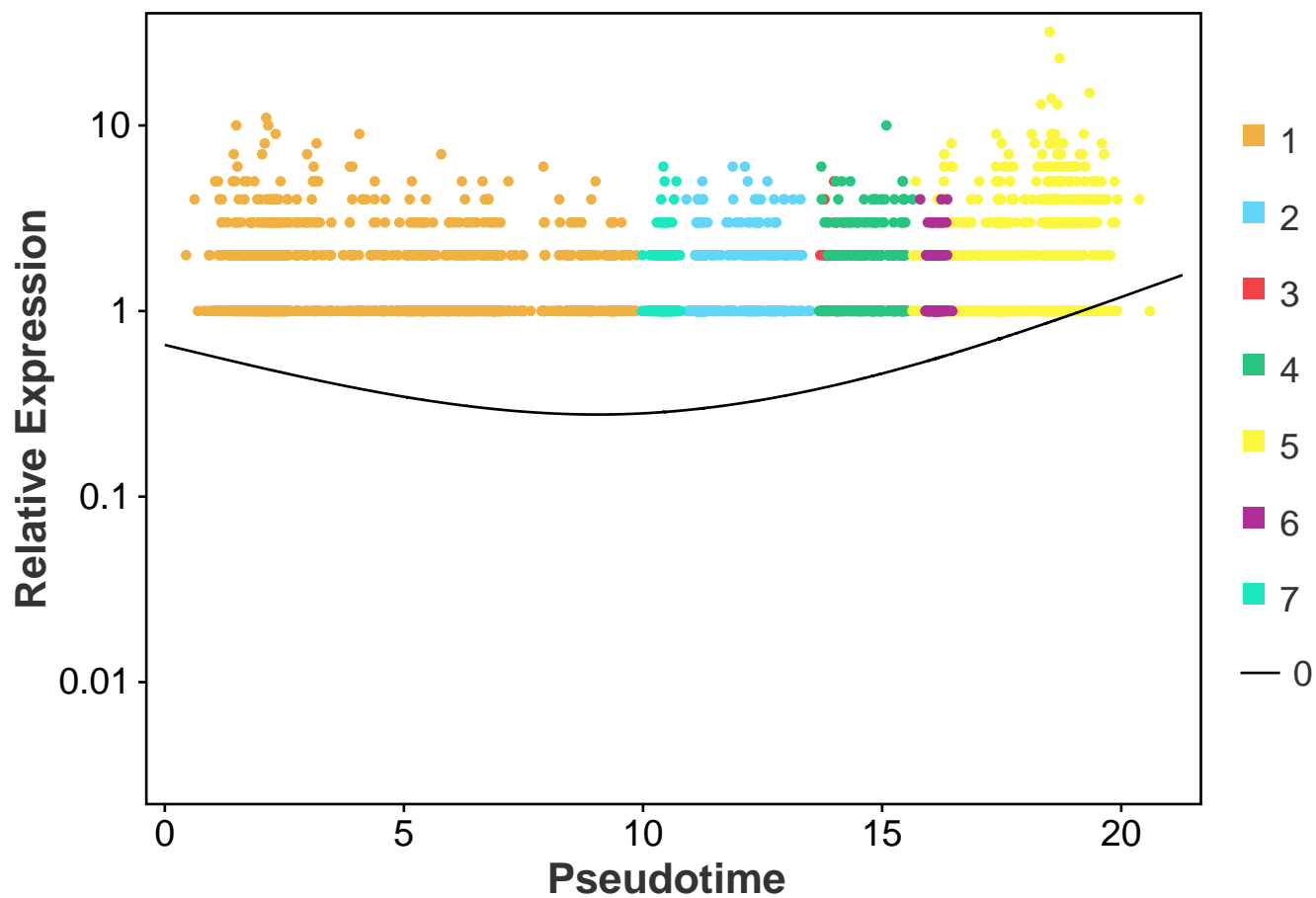

Supplement: Supplementary Figure 10 — Scatter plot of the expression level of PCD-related gene DiRD21A at different states on the pseudotime line. Showing the trend of the DiRD21A expression along pseudotime trajectory during differentiation of the epidermal cells. A dot in the scatterplot represents a single nucleus. The curve is a trend line. The X-axis was defined as “Pseudotime” and Y-axis was defined as “Relative expression” by log10 (exp + 1). [file Image_10.pdf]

# DiLSD1

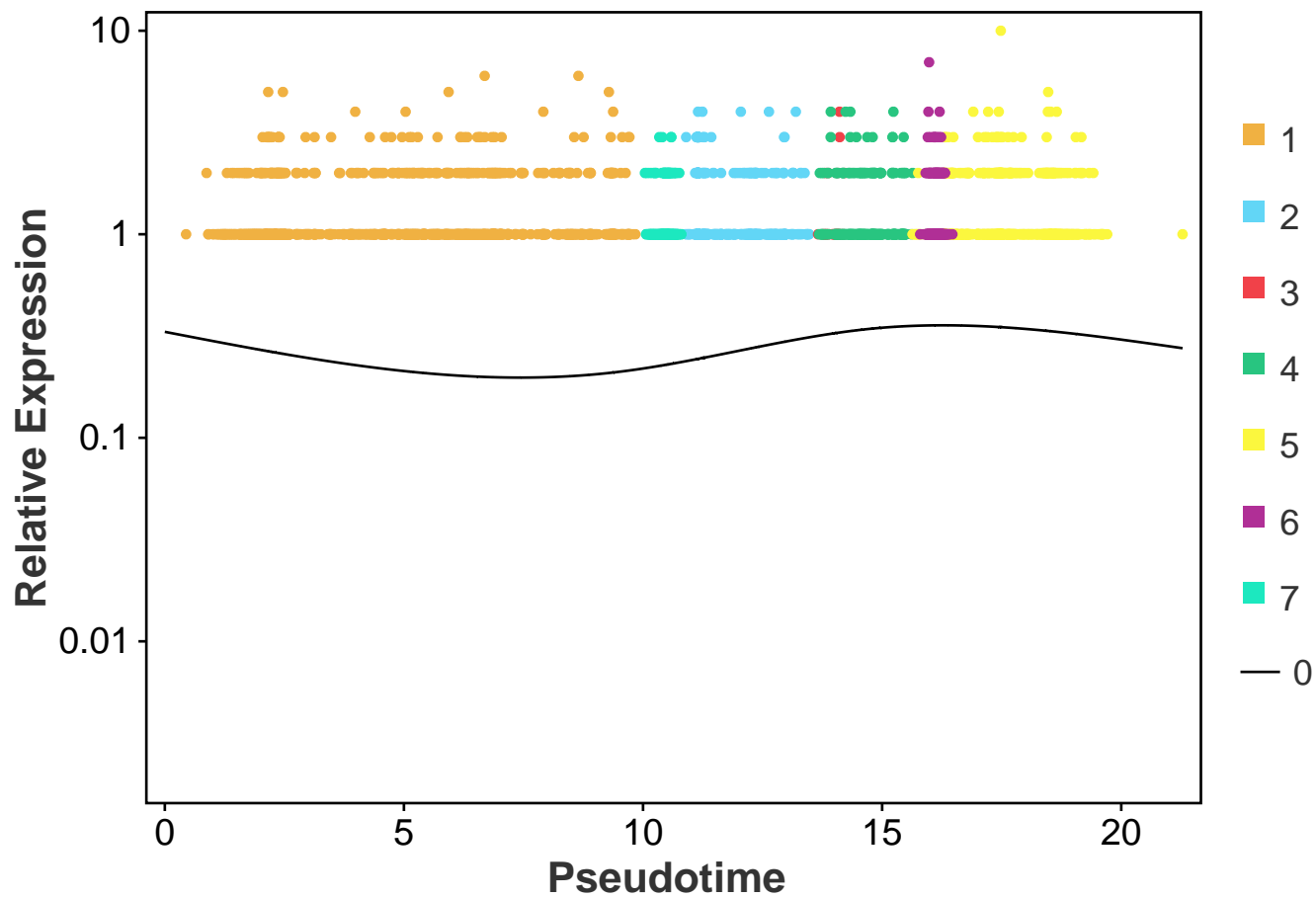

Supplement: Supplementary Figure 11 — Scatter plot of the expression level of PCD-related gene DiLSD1 at different states on the pseudotime line. Showing the trend of the DiLSD1 expression along pseudotime trajectory during differentiation of the epidermal cells. A dot in the scatterplot represents a single nucleus. The curve is a trend line. The X-axis was defined as “Pseudotime” and Y-axis was defined as “Relative expression” by log10 (exp + 1). [file Image_11.pdf]
