# Supplementary material for: Full-length RNA sequencing and single-nucleus sequencing deciphers programmed cell death and developmental trajectories in laticiferous canals of Decaisnea insignis fruits
Source: Front Plant Sci. 2024 Aug 13;15:1446561. doi: 10.3389/fpls.2024.1446561 (PMC11369900; doi:10.3389/fpls.2024.1446561)
Supplement: Supplementary Figure 9 — The DEGs of pseudotime between different states. Gene expression heatmap for all states of epidermal cells. [file Image_9.pdf]

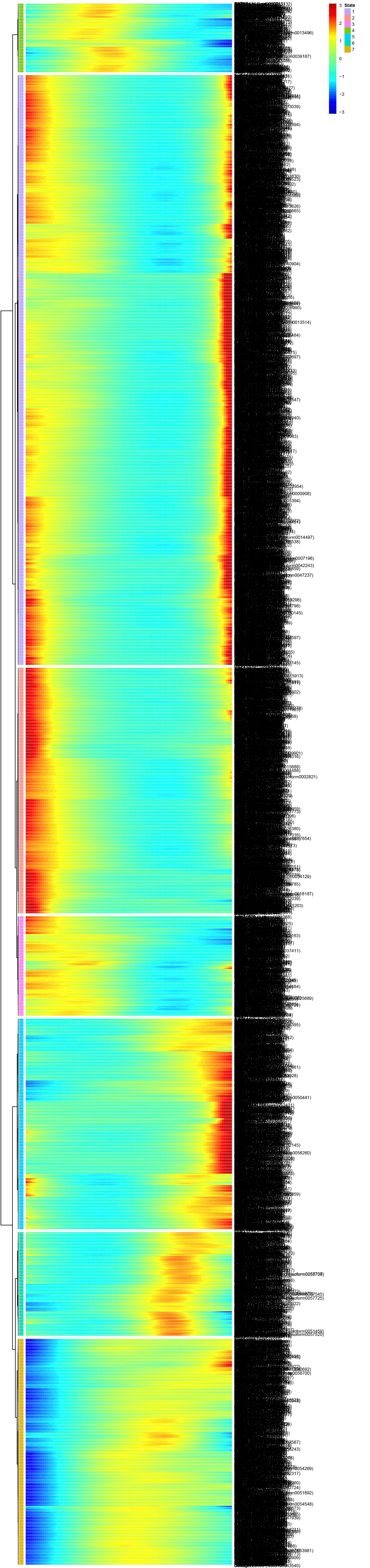

State
1
2
3
4
5
6
7
3
2
1
0
−1
−2
−3
